# Deep generative design of porous organic cages *via* a variational autoencoder†

**DOI:** 10.1039/d3dd00154g

**Published:** 2023-10-26

**Authors:** Jiajun Zhou, Austin Mroz, Kim E. Jelfs

**Affiliations:** a Department of Chemistry, Molecular Sciences Research Hub, Imperial College London White City Campus, Wood Lane London W12 0BZ UK k.jelfs@imperial.ac.uk

## Abstract

Porous organic cages (POCs) are a class of porous molecular materials characterised by their tunable, intrinsic porosity; this functional property makes them candidates for applications including guest storage and separation. Typically formed *via* dynamic covalent chemistry reactions from multifunctionalised molecular precursors, there is an enormous potential chemical space for POCs due to the fact they can be formed by combining two relatively small organic molecules, which themselves have an enormous chemical space. However, identifying suitable molecular precursors for POC formation is challenging, as POCs often lack shape persistence (the cage collapses upon solvent removal with loss of its cavity), thus losing a key functional property (porosity). Generative machine learning models have potential for targeted computational design of large functional molecular systems such as POCs. Here, we present a deep-learning-enabled generative model, Cage-VAE, for the targeted generation of shape-persistent POCs. We demonstrate the capacity of Cage-VAE to propose novel, shape-persistent POCs, *via* integration with multiple efficient sampling methods, including Bayesian optimisation and spherical linear interpolation.

## Abstract 2

Porous organic cages (POCs) are a class of porous molecular materials characterised by their tunable, intrinsic porosity; this functional property makes them candidates for applications including guest storage and separation.

## Graphical abstract

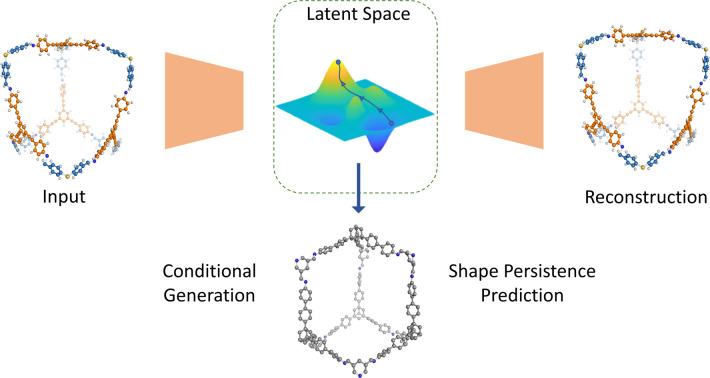

## 1 Introduction

**Fig. 1:**
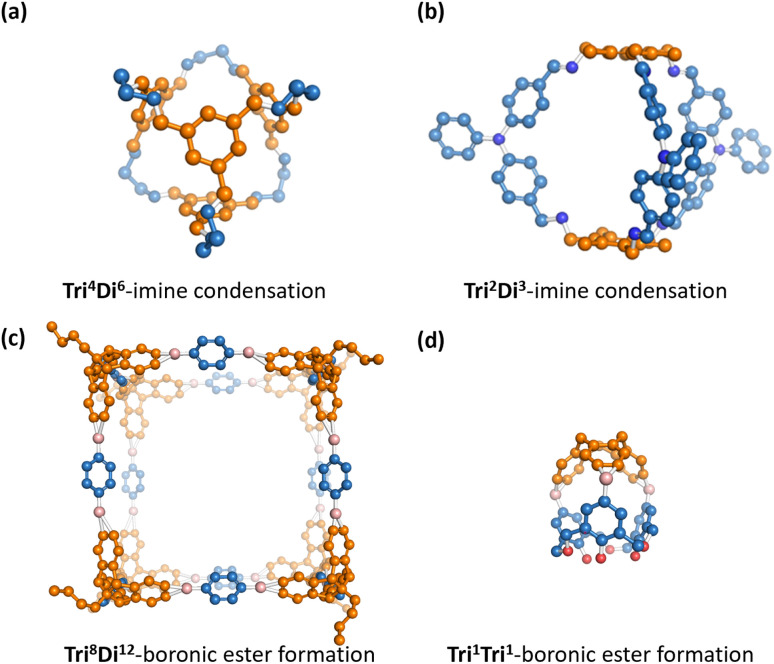
A collection of experimentally reported POCs^16–19^ with different topologies^12^ and formed from different reactions. Hydrogens are omitted for clarity. Vertex precursors (BB1) are shown in blue, while edge precursors (BB2) are depicted in orange.

Porous organic cages (POCs) are a class of molecular materials featuring an intrinsic cavity that can be accessed by several windows that allow bidirectional molecular passage.^1–3^ Compared to other porous materials, this intrinsic cavity is enclosed by the molecule itself so that the cavity is observable even in the form of a single discrete molecule. Due to the intrinsic void space in the solid state and the discrete form, POCs have potential in various applications such as molecular separations,^4,5^ sensing,^6^ proton conduction^7^ and catalysis.^8^ Examples of previously reported POCs are shown in Fig. 1. POCs are typically assembled from two molecular precursors. Component precursors with different stoichiometric ratios can form into POCs with different topologies. The intrinsic cavity of POCs is often not stable, and so the vast majority of hypothetical POCs are found to lose their cavity in the absence of solvent, a feature known as lacking “shape-persistence”.^9^ This loss of cavity results in a more dense, often non-porous amorphous phase, which decreases or, in some cases, eliminates both intrinsic and extrinsic porosity.^10^

The discovery of novel, shape-persistent POCs by conventional methods, where often only slight modifications are made to known POCs,^11^ is time-consuming and highly dependent on expert intuition and experience. The computational modelling of POC systems has become increasingly common, as it provides chemical knowledge of the new system before the experimental synthesis, and can significantly accelerate the discovery process. Current computational methods based on molecular dynamics (MD),^9,11,12^ density functional theory (DFT),^13^ and in-house software^14,15^ are often applied cooperatively for modelling POC structural features and their properties.

A range of rapidly evolving machine learning (ML) and deep learning (DL) approaches have been extended to multiple scientific areas with the development of improved computational hardware and capabilities. The development of ML and DL algorithms offers a solution for predictions of large-scale molecular systems involving high dimensional feature space, where the conventional computational approach becomes impractical.^20^ The applications of ML have covered a wide variety of topics within chemistry and material science such as drug discovery,^21,22^ retrosynthesis planning,^23^ and acceleration of theoretical calculations.^24^

In the discovery of POCs, ML has been applied to make predictions. For example, the prediction of the porosity of porous molecular materials based on crystallographic data.^25^ We have previously used ML models for property prediction, specifically POC shape persistence.^9^ To do so, we created a dataset of more than 60 000 POCs assembled *in silico* from a variety of di-, tri- and tetra-topic building blocks, using our supramolecular toolkit software, *stk*, which is a python library for modelling supramolecular chemistry.^14^ The random forest algorithm exhibited high accuracy in the discrimination of shape-persistent cages. Later on, an improved DL model, a graph neural network (GNN), was developed to predict shape persistence with the combination of molecular graph representations. Compared with the previous model, graph neural networks not only exhibited better performance, but improved model explicability.^26^

Discriminative ML models, aimed at modelling the conditional probability of the property of the given input data, are limited in the exploration of existing chemical space. Instead of learning the mappings from molecules to their properties, generative models can model the distribution of input molecules and depict the chemical space itself. Therefore, generative models are capable of generating synthetic molecules that have a similar distribution to the input molecules. As the generative model can produce results beyond the instances in the input, it is possible to use this approach to automatically expand the conventional chemical search space through *de novo* molecule generation without human intervention.^27,28^ In addition, the generative process can be subject to bias signals from one or several properties of interest, making it property-constrained. Contemporary generative models are typically based on DL due to the strong comprehensive performance of multi-layer neural networks.

A variational autoencoder (VAE)^29^ is an architecture to address the generative design problem with a high degree of flexibility in model construction and architecture, resulting in a highly modular approach. VAEs are capable of transforming molecules into continuous and compact representations in a latent space, where the patterns and structures in the collection of molecules can be captured and therefore generate new samples. Indeed, VAEs have shown promise for inorganic materials; there are recent models that underscore the significant progress of crystalline materials generation, both *via* VAEs^30^ and transformer architecture.^31^ VAEs have been applied to molecule generation and shown adaptability with multiple molecular representations ranging from one- to three-dimensions.^32–34^ In molecular science, Gómez-Bombarelli *et al.* first used a VAE with an external predictor to model small molecules and transfer them to continuous representations in the latent space, enabling the conditional exploration of molecules in the latent space *via* optimisations.^32^ Yao *et al.* then developed a VAE architecture capable of realising the conditional design of MOFs.^35^ However, a VAE for POCs has not yet been introduced. Indeed, there are distinct differences in the chemical composition of POCs and conventional crystalline, framework materials that impedes direct transfer to supramolecular materials; including, topological differences, which impacts feature selection.

Considering the modelling of a large chemical system such as a POC, the design of molecular representations requires careful consideration. Lower-dimensional representations, though easier to generate, are not able to retain sufficient structural information to fully describe POCs compared to their small molecule components. The three-dimensional conformation of a POC is not able to be approximated by a single SMILES string representation. In our previous study, POCs were decomposed into molecular fingerprints of precursors for ML predictions.^9^ However, this representation is non-recoverable to the original molecule and therefore it cannot be used in generative modelling. Thus, a new combinatorial representation that integrates both structural features of the precursor components and entire cage molecules needed to be developed. The strategy has recently been proven successful in the molecular design of several reticular frameworks, including metal–organic frameworks (MOFs) and zeolites. MOFs have been decomposed to multiple components, including sequential and one-hot representations, which were fed separately to the VAE-based model with multiple encoder–decoder pairs.^35^ In the design of zeolites, each unit lattice was represented by a combination of three, three-dimensional representations: the silicon grid, the oxygen grid, and the energy grid, and adopted in a Generative Adversarial Network (GAN).^36^

In this study, we have developed a deep generative model, Cage-VAE, based on the work of Gómez-Bombarelli *et al.*^32^ and Yao *et al.*,^35^ but specialised for the design of POCs; here, the decomposition of POCs and the target property necessitates modifications to the model architecture. Our model is able to generate novel, valid POCs with the **Tri**^**4**^**Di**^**6**^ topology that are shape-persistent. In addition, a combinatorial encoding system based on POC components, precursors and reactions, was developed to describe the structural and topological features of POCs, showing potential in the efficient representation of large molecular systems. The model architecture is transferable to the generation of other types of cage molecules that exhibit various properties of interest, such as metal–organic cages. The dataset and model are available at https://github.com/JiajunZhou96/Cage-VAE.

## 2 Methods

### 2.1 Dataset construction

**Fig. 2:**
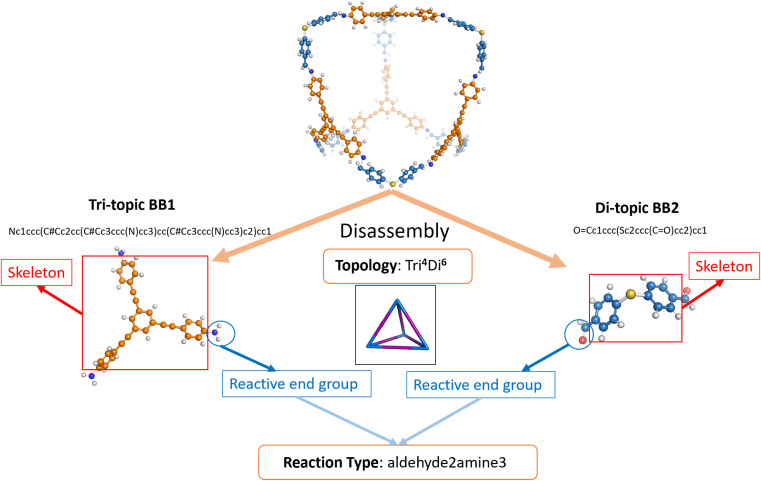
Schematic representation of the cage disassembly process in the dataset using a **Tri**^**4**^**Di**^**6**^ cage as an example. The tri-topic BB1 has *C*_3_ symmetry and three reactive end functional groups while the di-topic BB2 has *C*_2_ symmetry and two reactive end functional groups. The notation of the reaction type “aldehyde2amine3” indicates that the reaction is imine condensation. The tri-topic BB1 with three amine reactive end groups and di-topic BB2 with two aldehyde reactive end groups would react to form a cage.

The dataset of POCs used for this research was curated based on our previous works.^9,26^ In the dataset (referred to hereafter as the “original dataset”), 35 802 POCs were included and their shape persistence was labelled as either “collapsed” or “not collapsed” as per the original paper by Turcani *et al.*^9^ (see Table S1†). The authors used a two-step combinatorial method of computational calculation through MD simulations and in-house software *stk*^14^ and *pywindow*^15^ to determine the shape persistence. In the original work, POCs were labelled as “collapsed”, “non-collapsed”, or “undetermined”. To remove ambiguity and improve the robustness of our prediction of shape-persistent POCs, we relabelled all “undetermined” POCs as “collapsed”. The binary labelling also enables the exploration of interpolation as a strategy for conditional generation. POCs with **Tri**^**4**^**Di**^**6**^ topology assembled by four tri-topic building blocks (BB1s) and six di-topic building blocks (BB2s) were considered according to the notation developed by Santolini *et al.* (an example of a **Tri**^**4**^**Di**^**6**^ topology is shown in Fig. 2).^12^ To efficiently represent the complex structure, POCs were represented in a disassembled form consisting of two precursors and a text nomenclature denoting the topology. In this case, BB1 is the vertex precursor while BB2 is the edge precursor.

For each precursor (including both BB1 and BB2), the precursor skeleton and the reactive end functional groups were further separated. 117 di-topic precursor skeletons (BB2 skeletons) and 51 tri-topic precursor skeletons (BB1 skeletons) were included in the original dataset (see Table S4†). Several reaction regimes for constructing POCs were introduced in the dataset, including imine or amide condensation, and alkyne or alkene metathesis. Each reaction occupies the same proportion in the original dataset. To preserve the description of the assembled POC, the reactive end groups were removed from the skeleton SMILES representations and the resulting reaction type is obtained and appended to the end of the disassembled cage representation. The cage precursors stored in the dataset were represented by SMILES.^37^ The schematic representation of cage disassembly is shown in Fig. 2.

### 2.2 Molecular representations

The cage encoding was jointly described by the concatenation of the BB1 skeleton, BB2 skeleton and reaction type. The cage components are processed using different encoding methods; BB1 skeletons and reaction types are represented categorically, while BB2 skeletons are represented by SMILES strings. Not only does this maximise the efficiency of cage encoding and save computational resources, but it would be very challenging to balance the loss function if both BB1 and BB2 skeletons were represented by SMILES strings. As shown in Table S3,† the maximum length of tri-topic BB1 skeletons is nearly 50 characters longer than that of di-topic BB2 skeletons, a 100% increase. In addition, a large amount of BB2 skeletons lie in the low and medium-length regions (less than 50 characters long) leads to potential generations with higher validity. The character-level vocabulary of SMILES tokens (see Table S5†) was based on the combined set of the original and augmented BB2 skeletons constructed in Section 2.3. A special token “[Lr]” is used to denote the two reactive end functional groups sites in the BB2 skeleton; this was done to accommodate POC decomposition and improves our ability to determine the symmetry of generated BB2s. The “[sos]” and “[eos]” tokens were added to the beginning and end of all SMILES strings. SMILES strings were padded to the maximum length by the “[pad]” token. All BB2 SMILES strings that have lengths shorter than the maximum length were padded to the maximum length by the “[pad]” token. The two-character tokens were replaced with a single-character token (see Table S5†). All tokenised SMILES were converted to arrays of integers according to their index in the vocabulary. The size of the vocabulary including all special tokens is 30. The reaction type depicts the information of the supramolecular reaction responsible for the formation of cages, referencing reactive end functional groups in both precursors BB1 and BB2. As only 51 BB1 skeletons and 6 reactions appeared in the original and augmented dataset (shown in Tables S3 and S4†), the BB1 skeleton and reaction type are represented as categorical data and transformed into ordinal encodings. This also preserves the architecture of previously reported models,^35^ where the MOF components, which form the nodes/vertices of the framework materials, are represented categorically. Here, BB1 skeletons are the nodes/vertices of the **Tri**^**4**^**Di**^**6**^ POCs. Beyond this, truncating and representing BB1 skeletons categorically ensures a higher likelihood of generating feasible cages by preventing the accumulation of errors from potentially invalid SMILES strings.

### 2.3 Data augmentation

Training deep generative models requires large datasets (normally larger than 10^6^); the size of the original dataset in this work (35 802 POCs) was therefore insufficient. The size limitation originates from the highly limited instances of BB1 and BB2 in the original dataset. Due to the disassembled representation of POCs, one or several components can be augmented. Here, as BB2s are modelled using SMILES representations, it was naturally chosen to be the target of data augmentation. Therefore, a two-step combinatorial data augmentation strategy was used to boost the number of POCs. There is a potential benefit in choosing BB2 to be represented by SMILES from the perspective of data augmentation. As the di-topic precursors are located at the edges and therefore often almost linear, the POCs adopting augmented di-topic precursors for assembly are more likely to be chemically and topologically realistic. By data augmentation, the number of hypothetical POCs was increased to approximately 1.2 million, suitable for constructing a VAE generative model. Further details can be found in ESI Section 2.†

### 2.4 Variational autoencoder for *de novo* POC generations

**Fig. 3:**
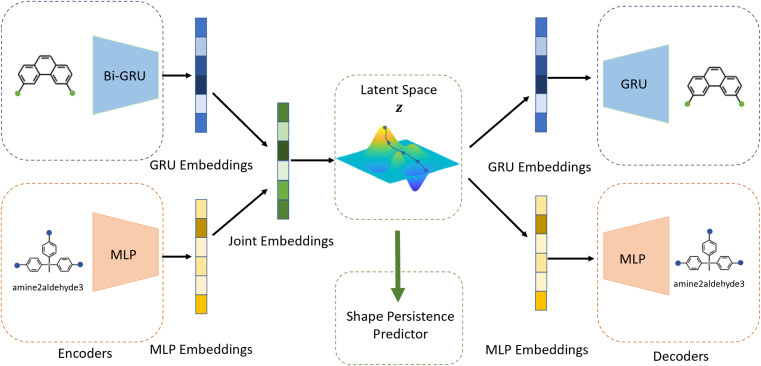
Schematic representation of Cage-VAE. Two encoder–decoder pairs cooperatively process information of the disassembled cage representation in a multi-component format. The latent representations of POCs are jointly constructed by the embedding of edge and vertex precursors (BB1 and BB2) and reactions encoded by GRU and MLP-based encoder modules. An additional MLP-based predictor establishes a target-oriented gradient to organise the latent space.

Building on previous works demonstrating VAEs^29^ for small molecule^32^ and MOF design,^35^ Cage-VAE, a multi-component VAE, was developed for POC generation. Cage-VAE was specifically developed for cage encoding. Each component in the POC encoding was processed in corresponding encoder–decoder pairs, modified to adopt the multi-component representation described above. Our Cage-VAE architecture enables the reconstruction of POC molecules from latent space and generates new POC molecules, as shown in Fig. 3. The training objective of a VAE is to maximise the evidence lower bound (ELBO)^29^ regularised by an adjustable parameter, *β*,^38^ according to:1
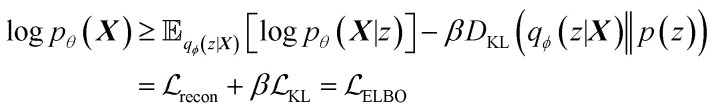
where the 
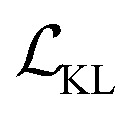
 term represents the Kullback–Leibler (KL) divergence between the prior distribution *p*(*z*) and the learnt posterior distribution, *q*_*ϕ*_(*z*|***X***). Here, the prior is assumed as the standard normal distribution. *β* is introduced to balance the expected reconstruction loss 
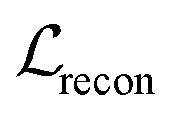
 and the KL term 
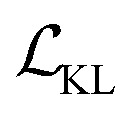
.^38^

The auto-regressive gated recurrent unit (GRU)^39^ was employed in both encoder and decoder architectures attributed to the sequence part processing for the BB2 skeletons of the cage encoding. To improve performance, we implement a bidirectional version of a GRU in the encoder for capturing the information of SMILES sequences in both the forward and backward directions. In this way, we can capture more structures in the sequence data, and, by extension, patterns from both directions. A single-directional GRU was used to decode SMILES sequences from the latent space. The BB1s and reaction types of the cage assembly are processed as combined vectors in an encoder–decoder pair using multi-layer perceptrons (MLP). It should be noted that the generation of either BB1 skeletons or reaction types is not beyond the existing categories in the original and augmented datasets due to the use of ordinal encodings. Encoders are responsible for jointly encoding components of cages to the continuous latent space **z**, and decoders reconstruct corresponding cage components from the latent space. The size of the hidden state of encoders is 256, while the size of the hidden state of decoders is 384. All encoders and decoders have a dropout rate of 0.25. The latent space has a size of 128. The shape-persistence predictor has a dropout rate of 0.5. A property predictor based on an MLP was then coupled to the VAE to predict the property from the latent space. The VAE was jointly trained with the property predictor to impose a property-based bias on the distribution of the embedded cages in the latent space.

The resulting multi-component loss function for training can then be represented as:2
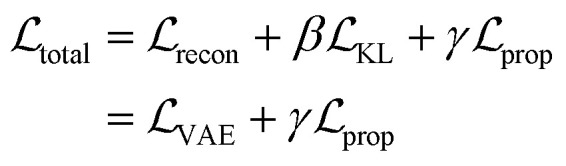
where the reconstruction loss of cages, 
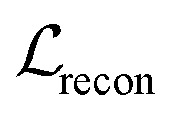
, is the weighted sum of the reconstruction loss term of the BB1 skeleton, 
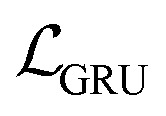
, and a reconstruction loss term of the BB2 skeleton and the reaction type, 
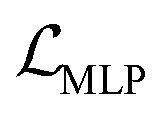
. 
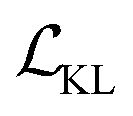
 is a KL divergence term regularised by an adjustable parameter, *β*.^38^ The combination of 
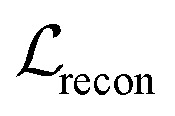
 and 
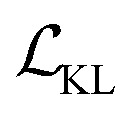
 terms form the evidence lower bound (ELBO), defined as the loss term for the VAE training. 
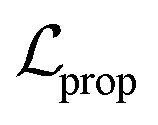
 is the loss for the training of the property predictor. The balance of the VAE and property predictor during the training is modified by *γ*. Due to the limited size of labelled data, the masked form of the predictive loss, 
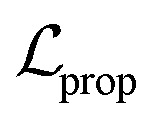
, was used to enable the semi-supervised training of the property predictor on labelled data only.

#### 2.4.1 Training schedulers

The naïve training of VAEs consisting of an auto-regressive decoding process often causes the KL vanishing, leading to the poor capture of meaningful information in the input data. As explained by Fu *et al.*,^40^ KL vanishing occurs due to an improper way of sequence generation, caused by the generation process relying only on the local context in the decoder while ignoring global features in the VAE. To deal with this issue, a cyclic annealing scheduler was used to adjust the value of *β* to direct the model to converge towards the training objective and reduce the effect of KL vanishing.^40^

We also applied schedulers on other loss terms. A linear scheduler was applied to the loss component 
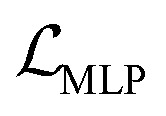
 for the reconstruction of BB1 and reaction type within the reconstruction term 
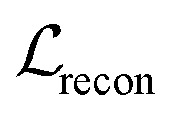
, inducing a monotonic increase from 0 to 1. The same scheduler was also applied to the property predictor. Our intention in using these two training schedulers was to enable the model to prioritise the reconstruction of the sequence representation of POCs during the initial stages of training, and gradually shift toward the reconstruction of the entire molecule and the organisation of the latent space as the training proceeded.

#### 2.4.2 Model training

To train the Cage-VAE, 90.0% and 99.8% POCs from the original (32 221) and augmented datasets (1 190 304) (see Table S2†) respectively are randomly selected as the training set, then mixed and imported for training. The data in the training set is maximised by this greedy splitting as the remaining data used in the test set is adequate, diverse and representative to serve the purpose of validating model performances. The remaining 3581 POCs from the original dataset were used as the test set to evaluate the loss and the accuracy of the prediction of the shape-persistence upon training. The remaining 2386 POCs from the augmented dataset were used as the test set to inspect the reconstruction loss upon training.

Cage-VAE was constructed using PyTorch.^41^ For the model training, the cross-entropy loss is used for the reconstruction of all POC components: BB2 sequence, BB1 and reaction type. The binary cross-entropy loss is used for the property prediction. In the loss function described by eqn (2), parameter *β* before the KL term 
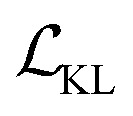
 was set to 0.0025. Parameter *γ* was set to 1.

The training process includes 100 epochs. The batch size is 64. During training, schedulers are used to stabilise the training process and improve the training performance. The cyclic scheduler was applied on the adjustable parameter *β* before the KL term 
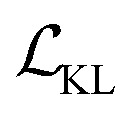
 to solve the KL vanishing issue.^40^ The cyclic scheduler starts from 0 and monotonically increases to *β* (0.0025) and maintains the maximum value for the remaining epochs of the cycle (see Fig. S9†). Five cycles are included in the training process. The monotonic linear schedulers were used to adjust both the 
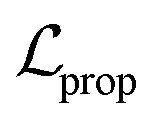
 and the component 
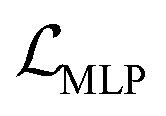
 for BB1 and reaction within the reconstruction term 
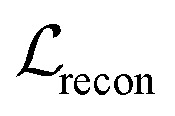
. Both linear schedulers increase gradually from 0 to 1 during the training. The Adam optimisation^42^ was used with a learning rate of 0.0001. The test loss curves of each term during the training are shown in Fig. S8.†

### 2.5 Cage sampling and optimisation

The design of novel and shape-persistent POCs relies on the generative capacity of the model. New POCs are represented as latent variables in the VAE latent space, with their shape persistence as the target property to be predicted by the auxiliary predictor directly from their latent variables. In the latent space of the VAE, the latent variables of POCs that are similar to each other are positioned closer within the learnt manifold formed by the continuous representations of POCs. In the structured latent space of the VAE, the resemblances of POCs are manifested in two factors: the similarity of molecular graph features depicted by SMILES and the similarity of the higher-level structure–activity relationship mapped by the auxiliary predictor.

We first explored the interpolation to be a conditional generation strategy. Generally, interpolation is not a typical conditional generation method because the attributes of the acquired molecules are not set explicitly, although the attributes of generated samples lying in the trajectory of interpolation are certainly influenced by these two endpoints. The transition of the attributes is also non-linear and the exact value of certain attributes can not be accurately estimated in the high dimensional latent space. However, the condition for the generated samples is a binary property here. When a proper threshold is set, the generated samples with a certain level of probability of shape persistence are considered eligible POCs. By ensuring that the trajectory of interpolation starts from a molecule with a high probability of shape persistence, the samples close to the starting molecule in the trajectory also have a high probability of shape persistence due to the structure in the latent space. The predicted probability of shape persistence is then compared with the predefined threshold to decrease the number of false positive samplings. We used spherical linear interpolation (*slerp*) as the default interpolation method (see ESI Section 5.5†). The probability threshold was set to 0.8 (the predicted probability of shape persistence should be at least 80%) to ensure a robust sampling result.

The conditional generation for POCs that are shape-persistent can alternatively be achieved using molecular optimisation. Bayesian optimisation is one of the most common strategies to navigate molecular optimisation in the latent space. The objective of Bayesian optimisation is to find the best POCs that meet the required condition depicted by the acquisition function. The exploration and exploitation of the Bayesian optimisation were balanced by adding a weighted regularisation term based on a standard normal distribution. We use the following acquisition function to achieve the latent representations of shape-persistent POCs:3
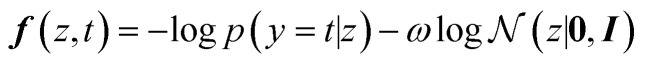
where *t* stands for the desired target value of the optimisation. Here, the target has a value of 0 in the labelling, representing cages that are shape-persistent. The term −log *p*(*y* = *t*|*z*) evaluates the negative log-likelihood when label *y* takes the target value *t* given the latent variable *z*. The term 
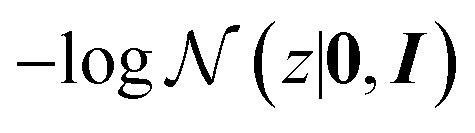
 is used for regularisation and balancing the exploration and exploitation using a parameter *ω*. By minimising the above acquisition function, the Bayesian optimisation is capable of obtaining the latent variable *z* corresponding to the cage molecules with an optimal probability of shape persistence.

The sampling and optimisation in the latent space inevitably result in invalid and unrealistic POCs due to the existence of dead regions where invalid SMILES are decoded.^32^ To reduce the effect of this issue, generation strategies can be concatenated with a filter. The filter is a flexible module designed based on simple heuristics towards structural and graphical features of cage components to validate generated POCs with the expense of minimal computational resources. The filter evaluates the generated POC in the order of validity, novelty, precursor validity, the number of reaction sites and symmetry. When a generated molecule fails to pass the filter, the current molecule is discarded and a signal is sent back to re-initiate a new cycle of generation. Therefore, a simple feedback loop was created to effectively alleviate the occasional sampling of problematic POCs.

The validation of generated POCs was carried out using molecular dynamics (MD) simulations by our previously employed cage modelling pipeline.^9,26^ The entire cage assembled by the precursors is generated and geometry optimised using the OPLS3 (ref. 43) forcefield. High-temperature MD simulations were then applied to search for the lowest energy conformations of cage molecules by sampling the potential energy surface of the cage conformation (700 K temperature for 2 ns after 100 ps equilibrium time). 50 conformers were sampled evenly along the MD trajectory and geometry optimised. The features of the lowest energy structure, cavity size, window diameter and the number of windows were calculated using *pywindow*^15^ and manually inspected to determine if the POC was shape-persistent.

## 3 Results and discussion

### 3.1 Evaluations *via* random sampling

**Fig. 4:**
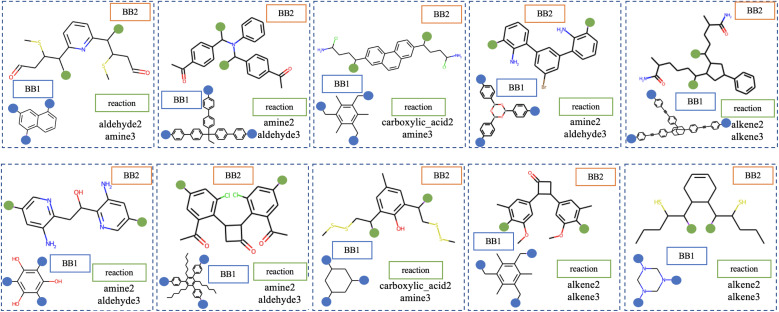
Visualisation of POCs in disassembled representations obtained *via* random sampling of latent space. The orange and blue coloured circle denotes sites for reactive functional groups in BB2 and BB1 skeletons, respectively.

The performance of generative models is assessed by sampling molecules from the latent space for the decoder to convert into a readable representation; random sampling is commonly used for this.^29^ Once sampled, it must be determined whether the sampled molecule is reasonable. For Cage-VAE, we represent cages in a deconstructed form and Fig. 4 shows several randomly sampled deconstructed cage molecules. From this, we observe variations in each of the three components of the cage encodings; BB1, BB2 and reaction types. The generated BB2 skeletons highly resemble the molecules in the training set.

The quality of generated molecules from corresponding latent variables is evaluated by several metrics, including common benchmarks such as validity, novelty and uniqueness and specific metrics designed for cages, such as precursor validity and symmetry (see ESI Section 5.1† for full definitions of these metrics). First, the validity of the generated molecules should be the top priority. Recall that in this model, Cage-VAE is designed to focus on the generation of BB2 skeletons as a component of disassembled cage representations and generate other components from finite sets. Therefore, the validity of POCs can be simplified as the validity of generated BB2 skeletons. In practice, this is achieved by determining whether the generated SMILES representation is syntactically and semantically valid enough to construct a molecular graph. To quantify validity, we sampled 1000 latent variables randomly; the validity of the decoded molecules reaches 0.917, as shown in Table 1. This indicates that our model effectively captures basic chemical rules without prior knowledge. It also indicates that the latent space is even and smooth to be decoded to valid SMILES strings.

**Table 1:** Evaluations of generated molecules upon random sampling. Here, the novelty of a generation is compared for both the original and the combined set of the original and augmented dataset

Evaluation metrics	Qualified rate
Validity	0.930
Novelty(original) + validity	0.924
Novelty(original + augmented) + validity	0.906
Uniqueness + validity	0.930
Precursor validity + validity	0.917
Symmetry + precursor validity + validity	0.654

Next, we independently consider novelty with respect to the original dataset and the combined set of the original and augmented dataset. We only consider the novelty of generated molecules that are also valid, in other words, validity is conditioned. Both the original and combined sets have novelties with very promising results of ∼0.900. When we consider the novelty of generated molecules against the larger combined dataset, we observe only a small decrease (0.906) as compared to the novelty of the original dataset. These large novelty scores demonstrate that Cage-VAE is capable of generating a large number of novel POCs from the latent space.

Subsequently, we consider the uniqueness of the generated POCs; this refers to the percentage of valid molecules that only appear once in a generation batch. POCs that appear multiple times are only counted once. We find that 0.930 of the valid, randomly sampled molecules are unique; this large value demonstrates that latent variables do not overlap and that dissimilar cages are located at different locations within the latent space. It also indicates that a more diverse distribution of valid cages is established through the generative model. The above metrics jointly indicate that our model can effectively extend the chemical search space of POCs under valid chemical rules.

The normal metrics to assess the performance of molecular generation are not comprehensive enough. Therefore, further metrics were included to assess extra performances of the POC generation, as shown in the last two rows of Table 1. Unlike the generation of small molecules, the generation of POCs is based on the disassembled cage representation. The SMILES strings of the BB2 skeletons are required to generate special molecules that are considered to be BB2 skeletons. The generated BB2 skeletons are featured to have two sites for reactive functional groups marked with a special token. To ensure that a proper BB2 skeleton was generated, precursor validity as an additional metric is introduced. Here the precursor validity is 0.917, indicating that the reaction sites reserved by the special token in precursors can be recognised and reconstructed during the training.

POCs included in this study possess highly symmetrical BB1 and BB2 precursors; this is an important prerequisite for POCs to be topologically described as **Tri**^**4**^**Di**^**6**^ by notations developed by Santolini *et al.* High *C*_*n*_ symmetry is preserved for both building blocks of POCs where *n* equals to the number of reactive end groups that participate in the cage assembly. Here, we hope that the precursors generated in the Cage-VAE exhibit high symmetry that resembles those samples in the training set. Though POCs with asymmetrical building blocks are reported to be achievable,^44^ the POCs with asymmetric building blocks are considered to have different distributions from the POCs with high symmetry building blocks, and potentially exist in large numbers of isomeric forms. For both generative and predictive modelling, the risk of error increases with the introduction of asymmetrical building blocks compared to when the input molecules are highly symmetrical. Therefore, symmetry is introduced as a metric to evaluate the proportion of generated building blocks that are symmetrical in a batch of sampling. The *C*_2_ molecular symmetry of BB2 skeletons is desirable, which can be approximated by graph symmetry. In practice, we determine whether two reaction sites in the graph of a single BB2 skeleton, depicted by its canonical SMILES string, have the same symmetry class. Therefore, the BB2 skeletons with their two reaction sites in the same symmetry class are evaluated to preserve high *C*_2_ symmetry or above the hierarchy of *C*_2_ symmetry and are considered as “symmetrical”. We observed this method can empirically align with our manual inspection of symmetrical BB2 skeletons.

The proportion of randomly generated molecules whose precursors are symmetrical is 0.654. This suggests that the model successfully recognises symmetry as a higher-level feature for the generation of BB2 precursors. The perception of symmetry is difficult as this feature is a completely recessive constraint. No information regarding molecular symmetry is explicitly input to the model during the training. In addition, the SMILES representation is lightweight to be used in generative modelling, but inherently weak in the depiction of molecular symmetry due to the depth-first tree traversal pattern.^45^ Thus, our model's capability to implicitly recognise and prioritise the concept of symmetry, despite the limitations of the SMILES representation, underscores its learning and generalisation capabilities.

We also constructed our model with BB2 skeletons represented by SELFIES.^46^ The results are shown in Table S7.† The inherent grammar constraints of the SELFIES string ensure a 100% validity in the validity of generated BB2 skeletons and high qualified rates in other general evaluation matrices. However, the SELFIES model shows a discernible decrease in the quality of the generation of BB2 skeletons with graph symmetry, manifesting as a 9.2% drop in performance compared to the SMILES model. It indicates that SMILES representation remains competitive for sequence-based generative models in task-specific adaptations.

### 3.2 Latent space organisation

The POCs were transformed into continuous latent vectors embedded in the latent space. Though VAE can organise the latent space by intrinsic characteristics of molecular representations, the latent space can also be effectively organised by properties mapped by an external predictor.^32,35^ Unlike the previous examples that used continuous targets as the signal, our property of interest, cage shape persistence, is a discrete classification. The MLP-based property predictor jointly trained with the VAE can achieve a test accuracy of 83.1% in shape persistence prediction. This suggests that the predictor captures the feature of shape persistence of molecules represented by latent vectors and therefore makes it possible for the predictor to correctly organise the latent space.

**Fig. 5:**
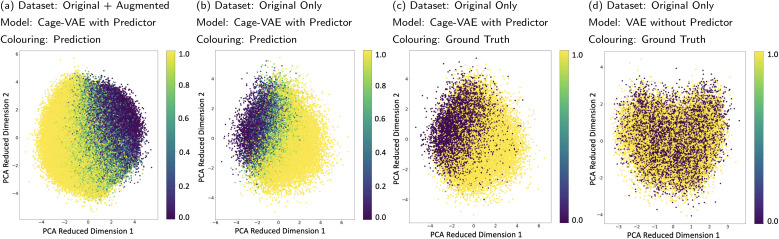
(a) PCA analysis of the latent space of Cage-VAE learnt by the joint training of the VAE and the property predictor using original and augmented datasets as the input of PCA. The probability mapped by the predictor is used to colour data points in the reduced dimensions. The colour bar shows the probability of the prediction on shape persistence where 0 and 1 are the lowest and highest probability of collapse, respectively. (b) The PCA analysis of the latent space of Cage-VAE using only the original datasets as the input of PCA. The probability mapped by the predictor is used to colour data points. (c) The PCA analysis of the latent space of Cage-VAE using only the original datasets as the input of PCA. The ground truth shape persistence label is used to colour data points. The two ends of the colour bar show the label of the prediction on shape persistence where 0 and 1 are the “non-collapse” and “collapse” property, respectively. (d) The PCA analysis of the latent space learnt by only training the VAE, using only the original datasets as the input of PCA. The ground truth shape persistence label is used to colour data points.

In order to inspect the learnt latent space, Principle Component Analysis (PCA) was used to visualise the position of POCs marked with properties in the compressed space as shown in Fig. 5a. Fig. 5a–c show the PCA performed on the latent space of Cage-VAE jointly trained with the predictor, while Fig. 5d shows the VAE trained without the predictor. In Fig. 5a, the latent vectors of both the original and augmented datasets are used for PCA and the probability of shape persistence prediction was used to mark data points in the PCA reduced dimension. A gradient of the probability of shape persistence mapped by the predictor is seen, clearly illustrating a smooth and continuous transition spectrum from the collapsed (light yellow) region to the non-collapsed region (dark violet). As discrete variables are disadvantageous in creating a gradient in the compressed latent space, we used a continuous representation of our discrete variable. Here, shape persistence is reflected as a probability that the POC will be shape-persistent. In both Fig. 5b and c, only latent vectors of the original dataset were used in the PCA. However, the probabilities of shape persistence predictions from the predictor and ground-truth labels were used in Fig. 5b and c, respectively. The similar pattern exhibited in these two latent spaces demonstrates that the predictor is accurate in mapping the cage latent vectors to their shape persistence feature and that the latent space is well organised. In Fig. 5d, the PCA analysis was performed on the latent space trained only by the VAE. The latent space shows no patterns with respect to the shape persistence, which reflects that the joint training of the VAE and predictor is effective for organising the latent space. To identify how the generated POCs compare with the training datasets, we plotted the generated molecules in Section 3.1 in the latent space depicted by Fig. 5a, detailed analysis can be found in Section 5.3 of the ESI.† From this, we observe that the latent representation of the generated POC samples reflects the candidates in the training dataset.

VAEs map input data into a distribution in the latent space, introducing stochasticity to the model and variations in decoded results. This feature allows VAEs to generate new samples. The reconstruction of a single POC can assess the model capacity of generation around the single latent point. The result of 1000 reconstructions of the same single POC is shown in Fig. S12.† The most frequent occurrence from the reconstruction is the original input molecule in ∼850 occasions, which indicates that the model successfully compressed POCs into the latent space. Multiple POC variations with structural similarity are also decoded from the latent representation, indicating that the model is capable of generating new samples based on the given molecule. In addition, with the increase of mean distance of the POC from the original input molecule, both the similarity between the decoded molecules and the original molecule and the occurrences are observed to decrease.

Next, interpolation was used to explore the latent space. When traversing from the initial to the final data points, novel POCs can be created across the trajectory of interpolation, which should demonstrate smooth transformations in their features. Therefore, the interpolated POCs share a certain degree of similarity and dissimilarity to both interpolation endpoints controlled by their positions on the interpolation trajectory.

**Fig. 6:**
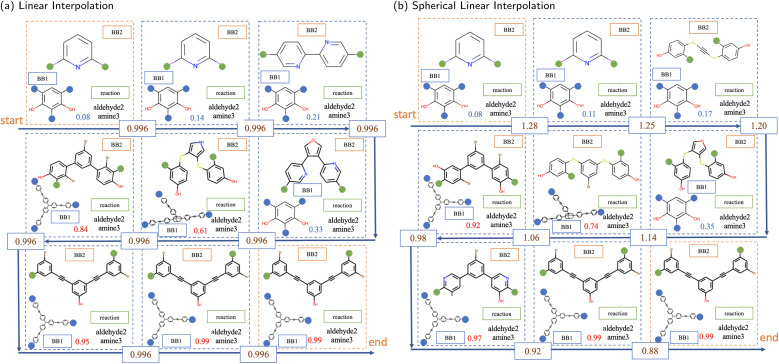
The sampled interpolations between the same pair of POCs from (a) linear interpolation and (b) spherical linear interpolation. The first and last POCs in each illustration are the starting and ending molecules of the interpolation. The Euclidean distance between embeddings of the starting and ending molecules is 7.98. The blue number in each unit containing a POC indicates the probability of collapse predicted by the predictor. The numbers on the arrows show the Euclidean distance between neighbouring latent vectors of POCs embedded in the latent space.

There are two interpolation methods commonly used to navigate the latent space in the application of generative modelling, linear (*lerp*) and spherical linear (*slerp*). The results of these two methods, with a fixed number of steps between the same pair of POCs, are shown in Fig. 6. In both interpolation methods, the transition from the initial to the final POCs is first found in the structural features of the BB2 skeletons. Slight changes or perturbations in the values in the latent vector can lead to the same decoding result. The structure of BB2 skeletons gradually changes from a relatively smaller molecule that has only a single benzene ring to three benzene rings. In the middle region of the interpolation trajectory, five-membered rings appear as intermediate states from linear chain backbones to benzene rings. It shows a smooth transition of structures from two benzene rings to three benzene rings. Another structural feature is that the length and complexity of BB2 backbones gradually increase from the initial to the final molecules. It is interesting that although only ordinal encodings were employed, BB1 skeletons also exhibit a structural transition from simpler to complex structures. In fact, BB1 skeletons and reactions are both observed to have transitions at a larger scale in the latent space. The approach to visualise the larger-scale transition can be simply to interpolate between POCs at a larger distance or interpolate across known transition boundaries.

In addition, the numbers in each unit, representing the probability of collapse of the cage molecule, have a monotonic increase in both interpolations. This demonstrates that the latent space is also effectively organised by the external shape persistence predictor. The current latent space is arranged cooperatively by features captured by both the VAE and the predictor. Compared to linear interpolation, spherical linear interpolation often results in an uneven sampling in the trajectory where the sample in the middle region is sparse, reflected by the probability value of shape persistence predictions. However, it may indicate that *slerp* finds it easier to obtain POCs with probability values close to the lower and upper limits in the prediction of shape persistence. In a binary predictive model, probabilities approaching the lower and upper limits typically denote a higher degree of confidence in the assigned label, which indicates that the predicted shape persistence of sampled novel POCs by *slerp* is potentially more robust.

### 3.3 Design of new POCs

The efficient sampling of the latent space of the generative model is the key to the design of new cage molecules with a desired property. Here, we would like to uncover new cage molecules that are shape-persistent. The well-trained gradient of the probability of shape persistence formed in the latent space enables the use of multiple gradient-based optimisation methods, such as Bayesian optimisation (BO)^32,47^ and reinforcement learning,^27,48^ that result in the conditional generation of non-collapsed cages. We use two different strategies, BO and interpolation, to demonstrate that our model is capable of combining different efficient sampling methods to realise the generation of new cage molecules.

**Fig. 7:**
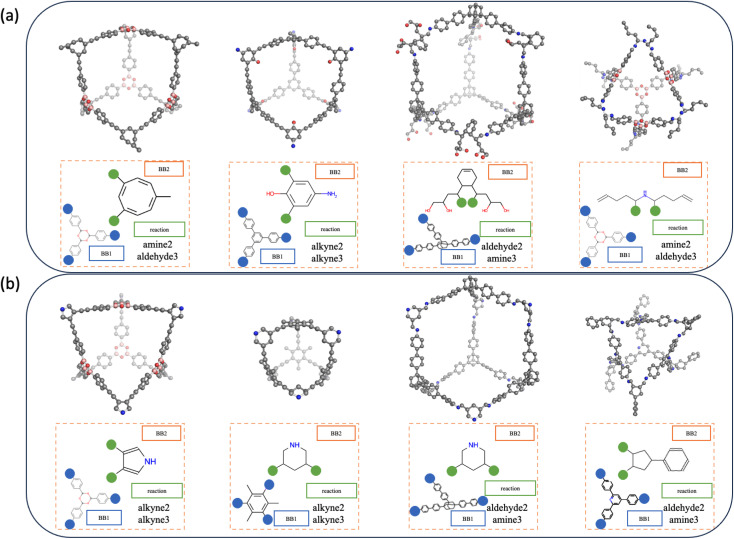
Conformers and disassembled representations of a selection of generated shape-persistent POCs *via* (a) Bayesian optimisation starting from a training set molecule and (b) *slerp* between randomly selected molecules in the training set that have a threshold of probability of prediction above 80%. All molecular conformations and shape persistence were validated by MD simulations.

For molecular optimisation, we used BO, which starts from a point in the latent space to gradually navigate to the point where the shape-persistent POCs are located. In order to increase the validity of sampled POCs and the efficiency of the convergence, the domain of each latent vector is restricted to the range enclosed by the minimum and maximum values of all training data, which is restricted but still allows us to interpolate and extrapolate from the training data. The POCs obtained by molecular optimisation are shown in Fig. 7a. These POCs feature unconventional backbones and side chains. However, the computational cost of BO is significantly larger than interpolation and the validity of the POC is normally compromised in exchange for the exploratory capability of this method.

Interpolation can be considered as an inbounds search method where the interpolation only traverses latent space enclosed by known molecules. In addition, due to the use of *slerp*, POCs with shape persistence generated by this interpolation method are more likely to lie on the learnt manifold. Therefore, the spherical linear interpolation method is efficient while not overly restricting the sampling. The POCs obtained by interpolation are shown in Fig. 7b. From the results, the interpolation can result in samples that are more likely to pass the filters from molecular validity to valid POC constructions and form shape-persistent POCs. Therefore, the traverse of the latent space within the bounds of the training dataset results in relatively “conventional” POCs that are observed to resemble known POC examples.

In both generation strategies, our Cage-VAE model shows a strong preference for forming shape-persistent POCs with alkyne metathesis. It can be attributed to the discovery that alkyne metathesis is most likely to form shape-persistent POCs, and these cages normally have high symmetry in the structures from MD simulations. Imine condensation involving amine and aldehyde functional groups has the second highest reaction occurrence, very close to alkyne metathesis. These results are in agreement with a previous study by Turcani *et al.*,^9^ where alkyne metathesis and imine condensation outperform other reactions for forming shape-persistent cages. The acquisition of shape-persistent cages formed by reactions that are not represented in Fig. 7 is also possible. However, to achieve the sampling of other reactions, the sampling methods need to be adjusted in order to be biased for specific reaction types, as they are not among the top targets to search for. In addition, by evaluating the cavity size of generated cages, both methods are capable of creating cages with varied cavity diameters typically ranging from 5 to 25 Å.

While Cage-VAE is the first generative model specialised in cage molecules, it also has limitations. The performance of the cage generation is robust, however, the predictions of shape-persistence may differ from the calculations obtained by MD simulations in certain cases. This can be traced back to the deviations in predictions in the shape persistence predictor, where the trained mappings from latent representations of cages to the property are not generalised to the synthetic cage samples far away from the current distributions due to the lack of labelling. Comparing the two strategies, molecular optimisation is observed to have more frequent erroneous predictions than the interpolation methods, as the molecules sampled using BO are more likely to have novel structural features and be away from the original distributions. In addition, the predictor included in our model is a general model designed for all reactions. Turcani *et al.* revealed discrepancies in predictive performances among cages assembled by different reactions.^9^ This is likely to be a source of error introduced to the general predictor as it needs to capture different patterns in reaction types and balance different features to obtain the overall best predictions. Finally, the presented VAE model is trained with minimal chemical knowledge and the shape persistence information is provided by the external predictor. In future work, the combination of more chemical knowledge explicitly or implicitly can be combined to fine tune the generative model and reorganise the latent space. Beyond this, and considering the inherent flexibility of POC topologies and structures, a diffusion model may present an interesting alternative architecture.

## 4 Conclusions

We have developed a VAE-based generative model, Cage-VAE, to realise the conditional design of shape-persistent POC molecules. The generative models are capable of reconstructing, inferencing and generating POCs. The generative model realises a smooth and continuous latent space jointly navigated by structural features and properties of interest and is capable of incorporating multiple sampling methods to freely explore and traverse through the massive chemical space of POCs. Our work provides a promising solution by DL for accelerating the discovery of POCs and this discovery theme can be transferred to other cage molecules and other porous materials. The dataset and model are available at https://github.com/JiajunZhou96/Cage-VAE.

## Data availability

All the data, code and models in this study is available in the Github repository. https://github.com/JiajunZhou96/Cage-VAE. Structures of all generated POCs in this study is available in *cage*/folder. To evaluate a trained model, use model_eval.py. To train cage-VAE from scratch, use training.py. Models trained in this study are available in the model/folder. Detailed descriptions and further information can be found in the above Github repository.

## Author contributions

J. Z. performed the calculations, developed the Cage-VAE code, and analysed the results. A. M. assisted in project design and execution. K. E. J. supervised the project. J. Z. wrote the manuscript and all authors contributed to the final version.

## Conflicts of interest

There are no conflicts of interest to declare.

## Supplementary Material

DD-002-D3DD00154G-s001
