# Supplementary material for: Deep generative design of porous organic cages via a variational autoencoder
Source: Digit Discov. 2023 Oct 26;2(6):1925–36. doi: 10.1039/d3dd00154g (PMC10695006; doi:10.1039/d3dd00154g)
Supplement: DD-002-D3DD00154G-s001 [file DD-002-D3DD00154G-s001.pdf]

Electronic Supplementary Material (ESI) for Digital Discovery.
This journal is © The Royal Society of Chemistry 2023

# Supporting Information: Deep Generative Design of Porous Organic Cages via a Variational Autoencoder

Jiajun Zhou, Austin Mroz, and Kim E. Jelfs*

*Department of Chemistry, Molecular Sciences Research Hub, Imperial College London, White City Campus, Wood Lane, London, W12 0BZ, U.K.*

E-mail: k.jelfs@imperial.ac.uk

# Contents


**1 Dataset Analysis** **S-3**

**2 Data Augmentation Strategy** **S-6**

**3 Model** **S-11**

3.1 Variational Autoencoder . . . . . . . . . . . . . . . . . . . . . . . . . . . . S-11

3.2 Auto-Regressive Model . . . . . . . . . . . . . . . . . . . . . . . . . . . . . S-13

3.3 SMILES vocabulary . . . . . . . . . . . . . . . . . . . . . . . . . . . . . . S-13

**4 Model Training Performance** **S-14**

**5 Evaluations** **S-16**

5.1 Evaluation Matrix . . . . . . . . . . . . . . . . . . . . . . . . . . . . . . . S-16

5.2 SELFIES Model performance . . . . . . . . . . . . . . . . . . . . . . . . . S-18

5.3 Analysis on the Generated POC Distribution . . . . . . . . . . . . . . . . . S-19

5.4 Reconstruction . . . . . . . . . . . . . . . . . . . . . . . . . . . . . . . . . S-22

5.5 Interpolation . . . . . . . . . . . . . . . . . . . . . . . . . . . . . . . . . . S-23

5.6 Filter . . . . . . . . . . . . . . . . . . . . . . . . . . . . . . . . . . . . . . S-23

**References** **S-24**

# 1 Dataset Analysis

Table S1: The number and percentage of POCs labeled by shape-persistence in the original dataset

| Shape-persistence | Number of POCs | Percentage |
|---|---|---|
| Collapsed | 23775 | 66.4% |
| Non-collapsed | 12027 | 33.6% |
| Total | 35802 | 100% |

Table S2: The number of porous organic cages in the supervised and unsupervised datasets and the training and test set splits.

| Dataset | Total | Training | Test |
|---|---|---|---|
| Original dataset (Supervised) | 35802 | 32221 | 3581 |
| Augmented dataset (Unsupervised) | 1192690 | 1190304 | 2386 |

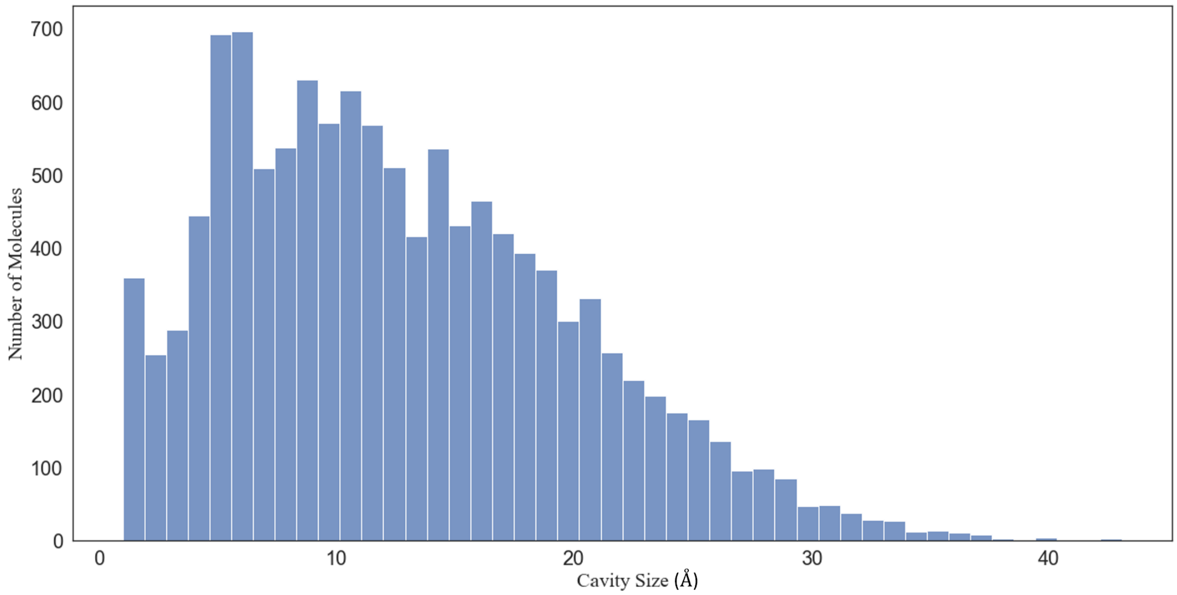


Figure S1: The cavity size (Å) distributions of non-collapsed POCs.

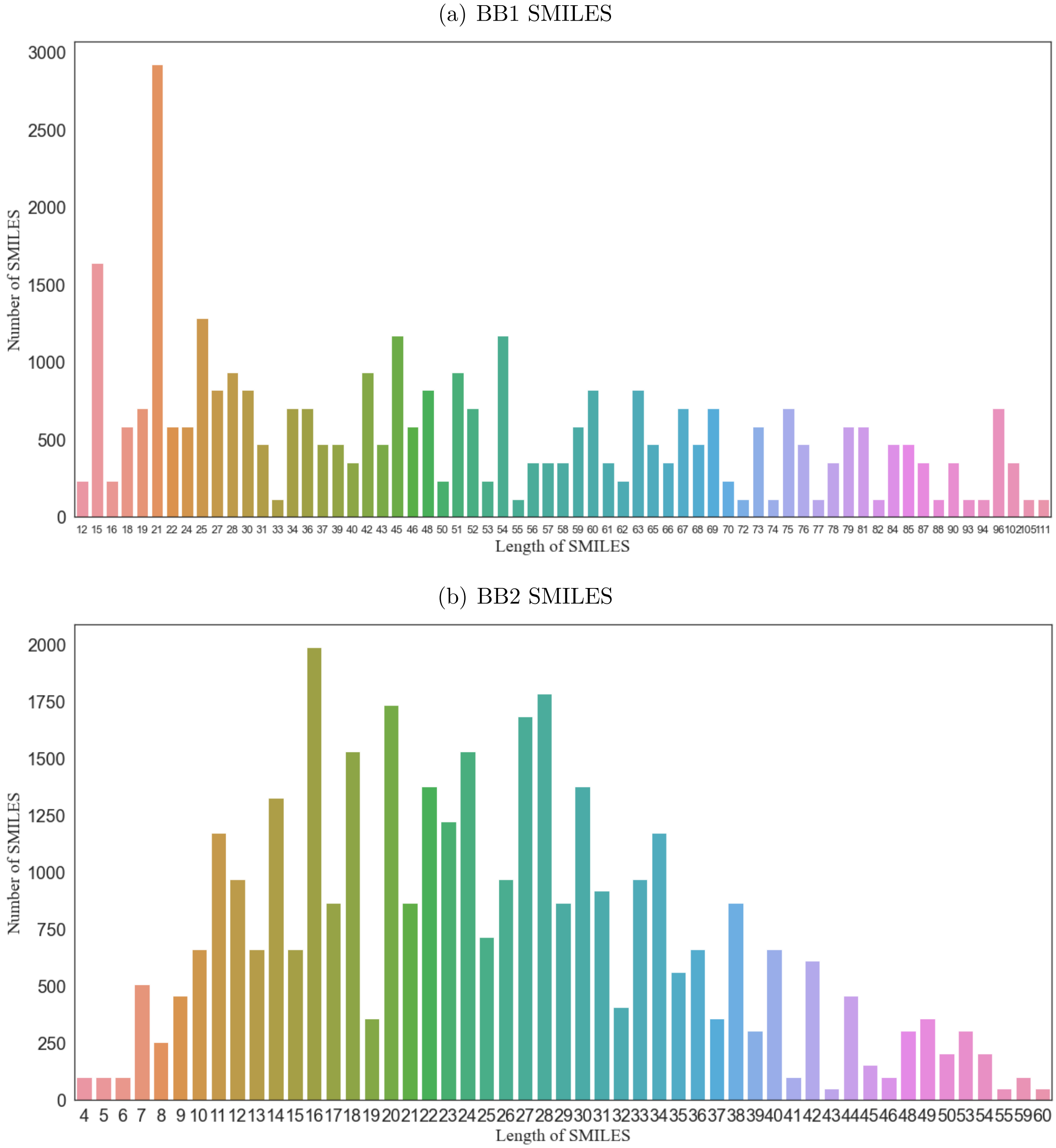


Figure S2: Length distribution of (a) BB1s and (b) BB2s in SMILES representation.

(a) BB1 skeleton SMILES

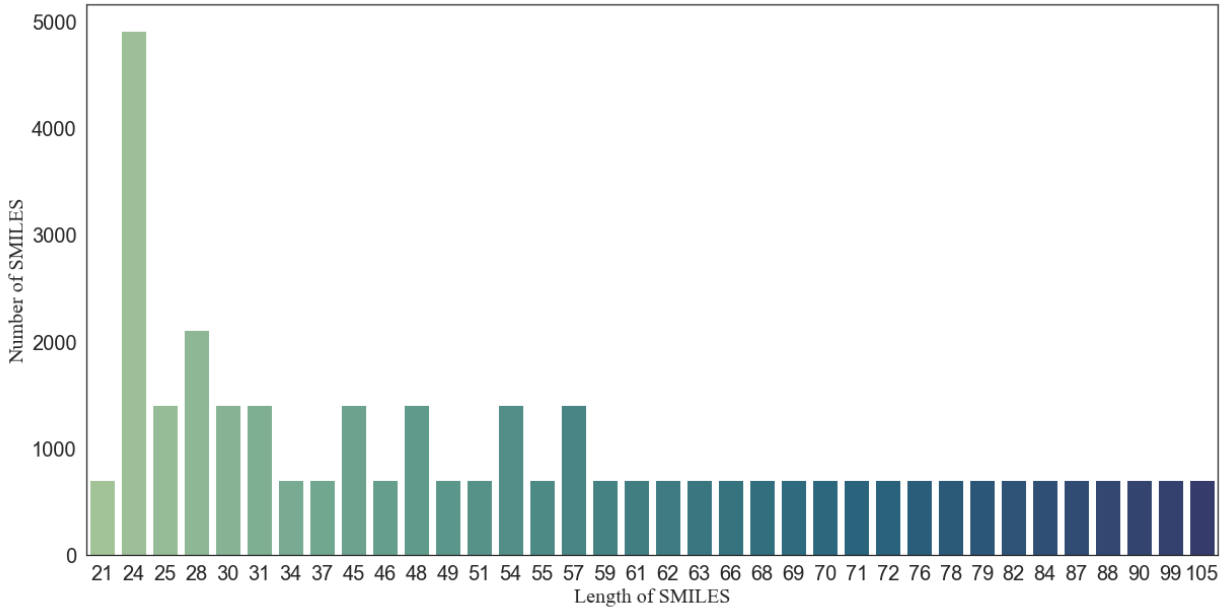


(b) BB2 skeleton SMILES

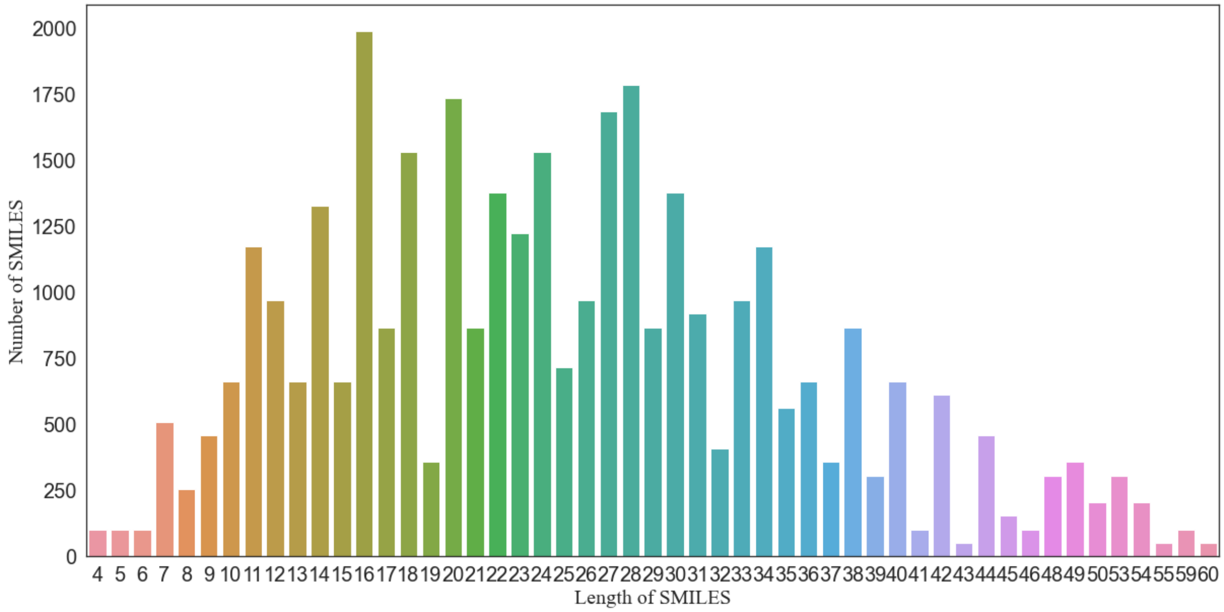


Figure S3: Length distribution (a) BB1 skeletons and (b) BB2 skeletons in SMILES representation.

Table S3: The number of POCs assembled by different reactions in the supervised and unsupervised dataset.

| Dataset | Nomenclature | Reactions | Number of POCs |
|---|---|---|---|
| **Original dataset** (35802) | aldehyde2amine3 | imine condensation | 5967 |
| | alkene2alkene3 | alkene metathesis | 5967 |
| | amine2aldehyde3 | imine condensation | 5967 |
| | amine2carboxylic_acid3 | amide condensation | 5967 |
| | carboxylic_acid2amine3 | amide condensation | 5967 |
| | alkyne2alkyne3 | alkyne metathesis | 5967 |
| **Augmented dataset** (1192690) | aldehyde2amine3 | imine condensation | 199345 |
| | alkene2alkene3 | alkene metathesis | 199119 |
| | amine2aldehyde3 | imine condensation | 198930 |
| | amine2carboxylic_acid3 | amide condensation | 198912 |
| | carboxylic_acid2amine3 | amide condensation | 198558 |
| | alkyne2alkyne3 | alkyne metathesis | 197826 |

Table S4: The number of categories of BB1 and BB2 skeletons and the minimum and maximum length of skeletons represented by SMILES string in the original dataset.

| Skeletons | Number of categories | Min length | Max length |
|---|---|---|---|
| BB1 | 51 | 21 | 105 |
| BB2 | 117 | 10 | 56 |

# 2 Data Augmentation Strategy

A two-step combinatorial method was developed to achieve BB2 augmentation. In the original dataset, BB2s exhibit high symmetry at a molecular level. This feature enables a convenient decomposition of a BB2 skeleton (with reactive end functional groups stripped from the BB2 backbone) to a precursor core and two subsequent linkers on both sides that are axially symmetrical with respect to the core. In the first step of data augmentation, the randomly chosen moieties of the core and linkers (a pair of identical linkers) were coupled to form a new precursor skeleton. The boundary between a core and a linker in the precursor skeleton is not strictly defined but is taken only for the convenience of deconstruction. In some cases, the moiety of core or linker is not present, which gives more flexibility in exploring augmentation possibilities. Therefore, precursor skeletons can be constructed by one of the following modes: $li + cr + li$, $cr$ itself and $li + li$. Subsequently, the entire BB2 can either

be mode (1).$R + li + cr + li + R$, (2).$R + cr + R$ or (3).$R + li + li + R$ (where $li$, $cr$, $R$ denote the linker, core and reactive end functional group, respectively). The examples of BB2 constructed by three strategies are shown in Fig. S4. In total, the most common 79 cores (excluding an empty element for the core-absent case) and 35 linkers (excluding an empty element for the linker-absent case) are selected as candidates to implement the random combination (shown in Fig. S5 and Fig. S6).

Besides the random combination, random functionalisation was performed as the second-step data augmentation method to further increase the number of available BB2s for virtual cage assembly. The random functionalisation was applied only to the linker moieties in BB2 skeletons to preserve the symmetry of the resulting POCs. Only one of the 20 functional groups was introduced in each functionalisation for a linker. The random functionalisation was designed to traverse all pairs of linkers in Fig. S7. Subsequently, the cores and generated linkers were combined to boost the number of BB2 skeletons using the same first-step random combination procedure described above. As a result of the two-step data augmentation, the number of POCs was raised to around 1.2 million and POCs were curated into a dataset (referred to as the "augmented dataset"). The size comparison of the original and augmented datasets can be found in Table S1 and Table S4.

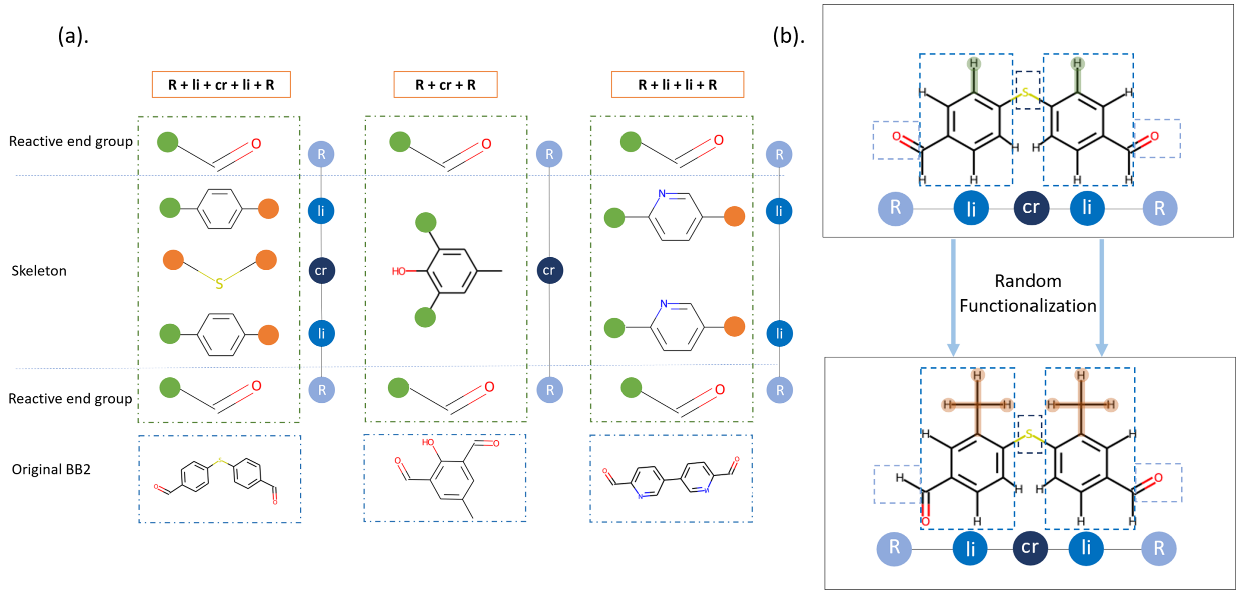


Figure S4: Schematic plot of the two-step data augmentation approach. a) Random combinations of sub-precursors as the first step. Three modes of BB2 construction are illustrated. From left to right: 1) $R + li + cr + li + R$, 2) $R + cr + R$ and 3) $R + li + li + R$. Where $li$, $cr$, $R$ denote the linker, core and reactive end group, respectively. The region of skeletons is specifically marked. Circles in the reactive end functional groups denote the connecting point. b). Random functionalization on the linker moieties in BB2 skeletons is the second step. The symmetry of BB2 skeletons is preserved after the augmentation.

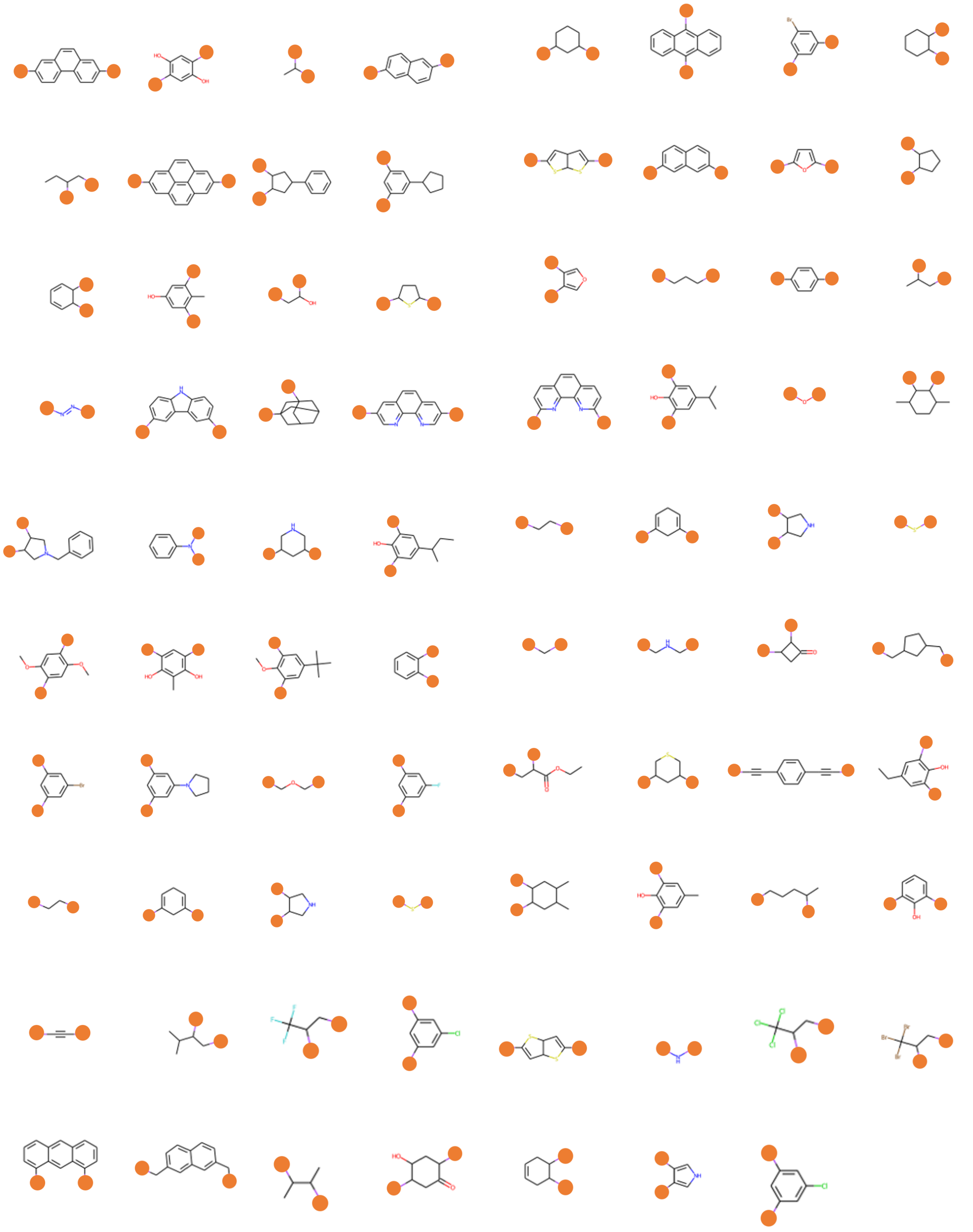

Figure S5: 79 core moieties for BB2 random combination (excluding an empty element in the core-absent case). The orange circles denote the connections between cores and linkers for mode (1).$R + li + cr + li + R$ or between cores and reactive end functional groups for mode (2).$R + cr + R$.

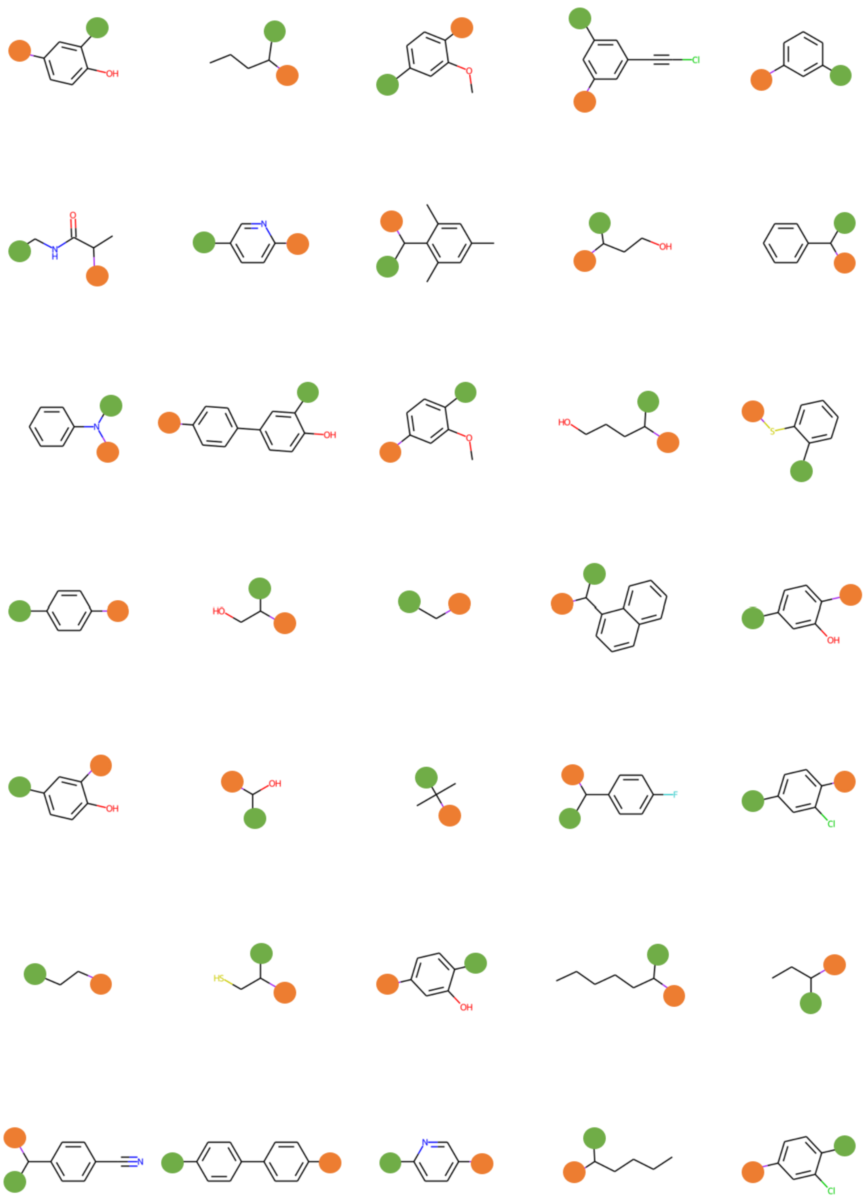


Figure S6: 35 Linker moieties for BB2 random combination (excluding an empty element in the linker-absent case). The orange circles denote the connections between cores and linkers for mode (1) $R + li + cr + li + R$ or between two linkers for mode (3) $R + li + li + R$. The green circles denote the connection between linkers and reactive end functional groups for mode (1) $R + li + cr + li + R$ and (3) $R + li + li + R$ or between cores and reactive end functional groups for mode (2) $R + cr + R$.

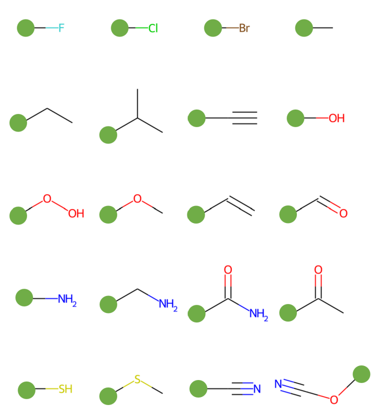


Figure S7: 20 Functional groups for BB2 random functionalization.

# 3 Model

## 3.1 Variational Autoencoder

A variational autoencoder introduced by Kingma and Welling[S1] is a generative model enabling the mapping of data $\boldsymbol{X}$ to a latent continuous variable $z$. The objective of this continuous latent variable model is to find a model distribution $q(z)$ based on characteristics of latent variable $z$ to approximate the true posterior $p(z|\boldsymbol{X})$. The encoder and decoder, usually modelled by deep neural networks, learn the approximate posterior $q_\phi(z|\boldsymbol{X})$ and the likelihood distribution $p_\theta(\boldsymbol{X}|z)$. The parameters $\phi$ and $\theta$ of the respective neural networks are learnt by maximising the evidence lower bound (ELBO):

$$\begin{aligned} logp_\theta(\boldsymbol{X}) &\geq \mathbb{E}_{q_\phi(z|\boldsymbol{X})}[logp_\theta(\boldsymbol{X}|z)] - D_{\boldsymbol{KL}}(q_\phi(z|\boldsymbol{X})||p(z)) \\ &= \mathcal{L}_{Recon} + \mathcal{L}_{KL} = \mathcal{L}_{ELBO} \end{aligned} \tag{1}$$

The $\mathcal{L}_{KL}$ term represents the Kullback-Leibler divergence between the prior distribution $p(z)$ and the learnt posterior $q_\phi(z|\boldsymbol{X})$. In practice, the prior is normally assumed as a multivariate Gaussian distribution.

As $\mathcal{L}_{KL}$ can be alternatively understood as a regularization term,[S2,S3] a hyperparameter $\beta$ is introduced to balance the extent of regularization during training,

$$\mathcal{L}_{ELBO} = \mathcal{L}_{Recon} + \beta\mathcal{L}_{KL} \tag{2}$$

$\beta$=1 is the original VAE proposed by Kingma and Welling.[S1] $\beta$=0 results in the degeneration of the VAE to a standard autoencoder (AE) for data reconstruction only and fail to explicitly encourage the formation of broad and even distributions over the latent space. By adjusting $\beta$ to a reasonable value in (0,1), the reconstruction quality of POCs can be improved while maintaining the generalisability of the VAE to unseen data.[S4,S5]

The VAE aims to generate the multi-component cage representation formed by BB1s, BB2s and reactions written as $\boldsymbol{X} = \{\boldsymbol{x_{bb2}}, \boldsymbol{x_{bb1}}, \boldsymbol{x_{rxt}}\}$ Where $\boldsymbol{x_{bb2}} = \{x_1, x_2, x_3, ..., x_t\}$ is a one-dimensional sequential molecular representation of predefined maximum length $n$ and the rest of components are categories. Three components of the cage representation are assumed conditionally independent given a common latent variable $z$. A valid POC data requires the presence of all three components. Each component was encoded by learnt deep neural networks $\boldsymbol{\phi} = \{\phi_1, \phi_2, \phi_3\}$ and decoded by $\boldsymbol{\theta} = \{\theta_1, \theta_2, \theta_3\}$. Therefore, the $\mathcal{L}_{recon}$ term can be split into two components $\mathcal{L}_{GRU}$ and $\mathcal{L}_{GRU}$ for the reconstruction of $\{\boldsymbol{x_{bb2}}\}$ and $\{\boldsymbol{x_{bb1}}, \boldsymbol{x_{rxt}}\}$.

The distribution of POCs over latent variable $z$ can be organised by the property of POCs by adding a predictor $q_\phi(y|z)$[S6] to enforce the property-oriented constraint.

$$\mathcal{L}_{prop} = \mathbb{E}_{(y,z)\in S_{supervised}}[-log q_\phi(y|z)] \tag{3}$$

Where the predictor takes data from the supervised dataset. $z$ in the supervised domain $z \in S_{supervised}$ can be obtained by mapping supervised data $\boldsymbol{X} \in S_{supervised}$ into latent space via the encoder $q_\phi(z|\boldsymbol{X})$.

Therefore, the multi-component loss function with adjusted magnitudes in terms can be written as:

$$\begin{aligned}\mathcal{L}_{total} &= \mathcal{L}_{GRU} + \mathcal{L}_{MLP} + \beta\mathcal{L}_{KL} + \gamma\mathcal{L}_{prop} \\ &= \mathcal{L}_{recon} + \beta\mathcal{L}_{KL} + \gamma\mathcal{L}_{prop} \\ &= \mathcal{L}_{VAE} + \gamma\mathcal{L}_{prop}\end{aligned} \tag{4}$$

## 3.2 Auto-Regressive Model

For the generation of sequences $\boldsymbol{x_{bb2}} = \{x_1, x_2, x_3, ..., x_t\}$, an auto-regressive encoder-decoder pair both based on gated recurrent units (GRU) is used,[S7] with the difference that the encoder employs the bi-directional architecture while the decoder is one-directional only. By incorporating an auto-regressive decoding process in VAE architecture, the generation of a token at time step $t$ is based on both the local context, i.e., all tokens generated in previous time steps $x_{<t}$, and global features, i.e., the latent variable $z$:[S3]

$$p_\theta(\boldsymbol{x_{bb2}}|z) = \prod_{t=1}^{t} p_\theta(x_t|x_{<t}, z) \tag{5}$$

For the generation of the rest of the cage representation, Multi-layer perceptrons are applied to the encoder-decoder pairs processing the category-represented components of the POC $\{\boldsymbol{x_{bb1}}, \boldsymbol{x_{rxt}}\}$ whose decoding process is solely dependent on the latent variable $z$.

In comparison, multi-layer perceptrons are applied to process the category-represented components of the POC $\{\boldsymbol{x_{bb1}}, \boldsymbol{x_{rxt}}\}$ whose decoding process is solely dependent on the latent variable $z$.

## 3.3 SMILES vocabulary

Table S5: Vocabulary of SMILES strings. The two-character tokens “Br” and “Cl” are replaced by single-character tokens “R” and “G”. The special integrated token “[Lr]” is converted to “X”. The functional tokens “[nop]”, “[sos]” and “[eos]” are replaced by single character tokens “$”, “¥” and “£”.

| **SMILES vocabulary** | | | | |
|---|---|---|---|---|
| $ | ¥ | £ | # | ( |
| ) | - | / | 1 | 2 |
| 3 | 4 | 5 | = | B |
| C | F | G | H | N |
| O | R | S | X | [ |
| ] | c | n | o | s |

# 4 Model Training Performance

Table S6: The evaluations of test set performances at the last epoch of the training process.

| **Evaluation matrix** | **Value** |
|---|---|
| Reconstruction Loss | 0.012 |
| Category Reconstruction Loss | 0.001 |
| KL Loss | 64.63 |
| Prop Loss | 0.364 |
| Prop Accuracy | 0.841 |

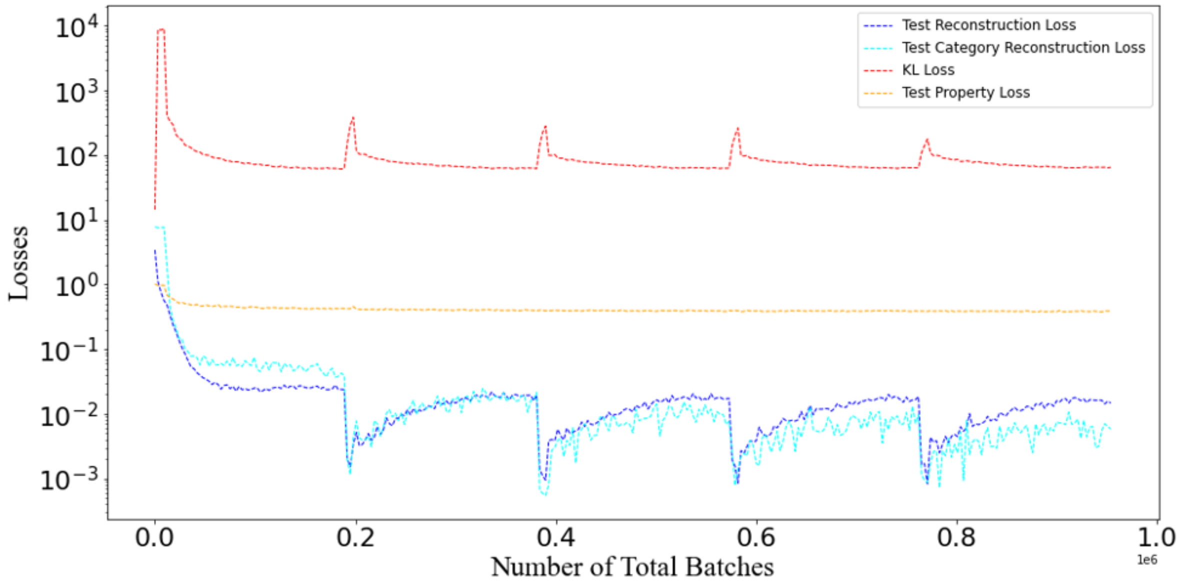


Figure S8: The logarithmic loss curves of all loss components on the fixed test set.

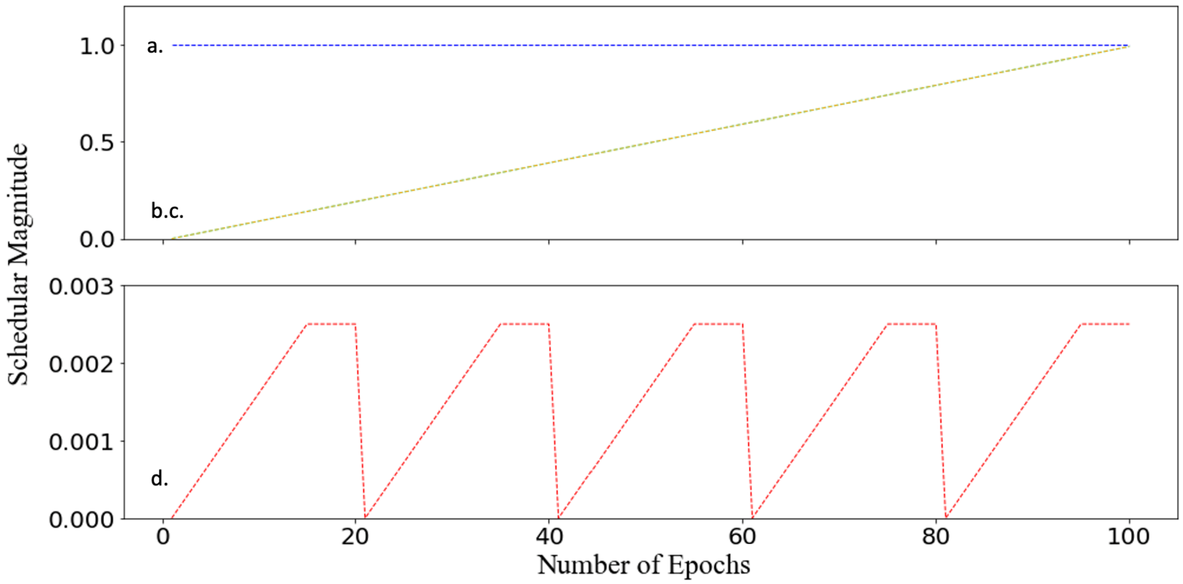


Figure S9: The magnitude of all schedulers during the training process. a). constant scheduler for sequence reconstruction loss term $\mathcal{L}_{GRU}$. b) and c). linear scheduler for category reconstruction $\mathcal{L}_{MLP}$ and property loss term $\mathcal{L}_{prop}$ and d). cyclic scheduler for KL divergence term $\mathcal{L}_{KL}$.

# 5 Evaluations

## 5.1 Evaluation Matrix

The random sampling of the latent vectors of POCs follows a standard normal distribution. The batch size is 1000 for the random sampling. The evaluation of the validity of generated POCs can be simplified to evaluate the validity of generated SMILES of BB2 skeletons. The validity of the SMILES was evaluated by RDKit.[S8] The equation of validity is shown in Equation 6 where $N$ denotes the number of molecules:

$$Validity = \frac{N_{Valid}}{N_{Batch}} \tag{6}$$

The novelty of molecules is compared with either the original dataset or the union set of the original and augmented datasets. The novelty should consider all three components of the POC. Novelty + Validity was evaluated by the following equation:

$$Novelty + Validity = \frac{N_{Novel}}{N_{Valid}} * Validity \tag{7}$$

In the calculation of uniqueness, molecules that appear only once and only the first occurrence of molecules that appear multiple times will be counted as unique molecules. Uniqueness + Validity can be calculated using a similar equation compared with Novelty + Validity:

$$Uniqueness + Validity = \frac{N_{Unique}}{N_{Valid}} * Validity \tag{8}$$

The POC is considered to have precursor validity with generated BB2 skeletons having precursor validity. The precursor validity evaluates the number of reaction sites denoted in generated BB2 skeletons. The calculation of Precursor Validity + Validity is:

$$Precursor\ Validity + Validity = \frac{N_{Precursor\,Valid}}{N_{Valid}} * Validity \tag{9}$$

Symmetry refers to the graph symmetry in our study. Similarly, the evaluation of the symmetry of POCs was simplified to evaluate the symmetry of generated BB2 skeletons. We used Open Babel[S9] to evaluate the graph symmetry of BB2 skeletons based on their canonical SMILES representations. In the $C_2$ symmetrical BB2 skeletons, the symmetry class of two reaction sites should be identical. Therefore, BB2 skeletons that have reaction sites with the same symmetry class are considered to pass the symmetry evaluation. In addition, symmetry should be considered for BB2 skeletons that pass the SMILES validity and precursor validity. Therefore, the Symmetry + Precursor Validity + Validity is calculated as:

$$\begin{aligned} & Symmetry + Precursor\ Validity + Validity \\ = & \frac{N_{Symmetry}}{N_{Precursor\ Valid}} * (Precursor\ Validity + Validity) \end{aligned} \tag{10}$$

## 5.2 SELFIES Model performance

We have also tested the model performance using SELFIES as the input. The maximum length of the SELFIES string was 50. Similar to the vocabulary of SMILES, the vocabulary of SELFIES tokens is constructed including four special tokens indicating the start of the sequence, the end of the sequence, the padding and the notation of the site of the reactive end functional group. The model was adapted to accept the SELFIES string input while preserving other conditions, including the dataset, dataset split ratio, overall model architectures, layer dimensions, training schedulers, the optimizer and all hyperparameters that do not affect the SELFIES adaption.

Table S7: Evaluations of generated molecules using the SELFIES and SMILES representation upon random sampling.

| **Evaluation metrics** | **Qualified Rate (SELFIES)** | **Qualified Rate (SMILES)** |
|---|---|---|
| Validity | 1.000 | 0.930 |
| Novelty(original) + Validity | 0.996 | 0.924 |
| Novelty(original + Augmented) + Validity | 0.974 | 0.906 |
| Uniqueness + Validity | 1.000 | 0.930 |
| Precursor Validity + Validity | 0.945 | 0.917 |
| Symmetry + Precursor Validity + Validity | 0.562 | 0.654 |

## 5.3 Analysis on the Generated POC Distribution

To identify how the generated POCs compare with the training datasets, we plotted the generated molecules in Section 3.1 in the latent space depicted by Fig. 5(a). As shown in Fig. S10, for each principal component dimension, we used Kernel Density Estimation (KDE) to assess the distribution of the data points. Here, the generated POCs have a similar distribution to the training set in both reduced dimensions, yet the distribution of generated POCs has a lower variance. This indicates that the generated POCs reflect the information in the training set, which includes both original and augmented POCs. We also compared the distribution of generated POCs to the original and the combined set (Original + Augmented), respectively. To quantify the difference between the generated samples and the POCs in the training set, we computed the KL divergence between datasets latent representation of the generated POC samples and the latent representation of the original and combined datasets. By comparison (shown in Table S8), the approximated KL divergence between the generated POCs and combined dataset is significantly smaller than the original dataset. Therefore, the generated POCs resemble the combined dataset than the original dataset.

Table S8: Estimated KL divergence between distribution of the latent representation of the generated POCs and distributions of the original or Combined set, independently.

| | **Original dataset** | **Combined dataset** |
|---|---|---|
| **Generated POC samples** | 62.29 | 9.97 |

To further illustrate the similarity between the generated samples and the training dataset, we used the combined Original + Augmented dataset to construct a PCA model and subsequently transformed 1000 randomly sampled generated POCs into the reduced PCA space, Figure S10(a). For clearer visualisation, we plotted 30000 training molecules from the combined set of Original + Augmented dataset and all 1000 generated POCs. The generated POCs scatter in the entire PCA space and separate clusters are not formed, Figure S10(b). This indicates that, i) the distribution of generated POCs is very similar to the training set,

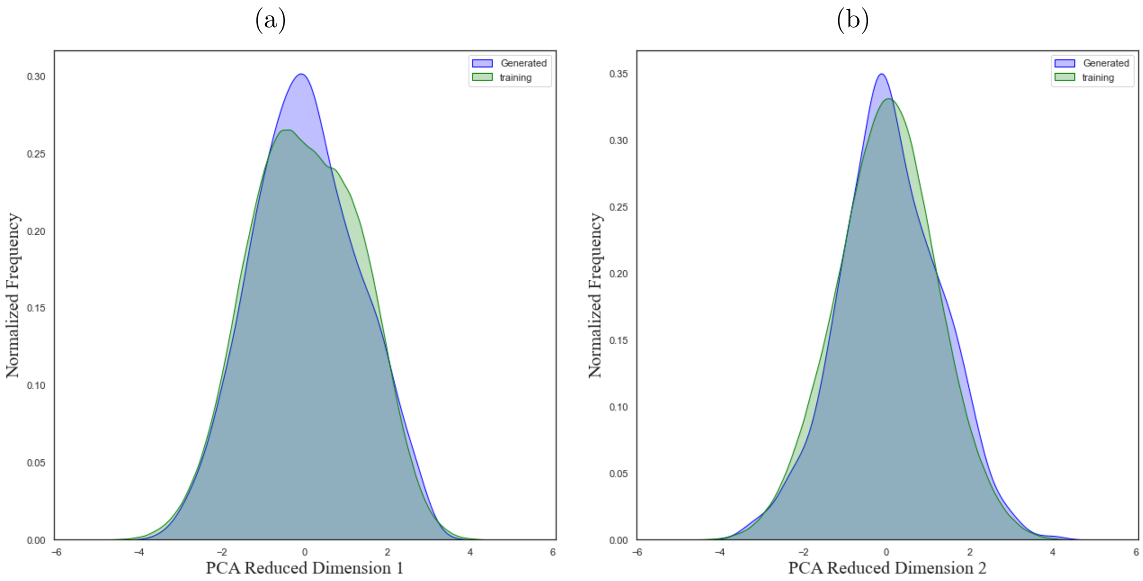


Figure S10: Kernel density estimate of the latent representation of generated samples and samples of the training data for a). principle component 1 and b) principle component 2 of the PCA.

and ii) Cage-VAE can capture the pattern of POCs in the training set and therefore generate valid POC instances.

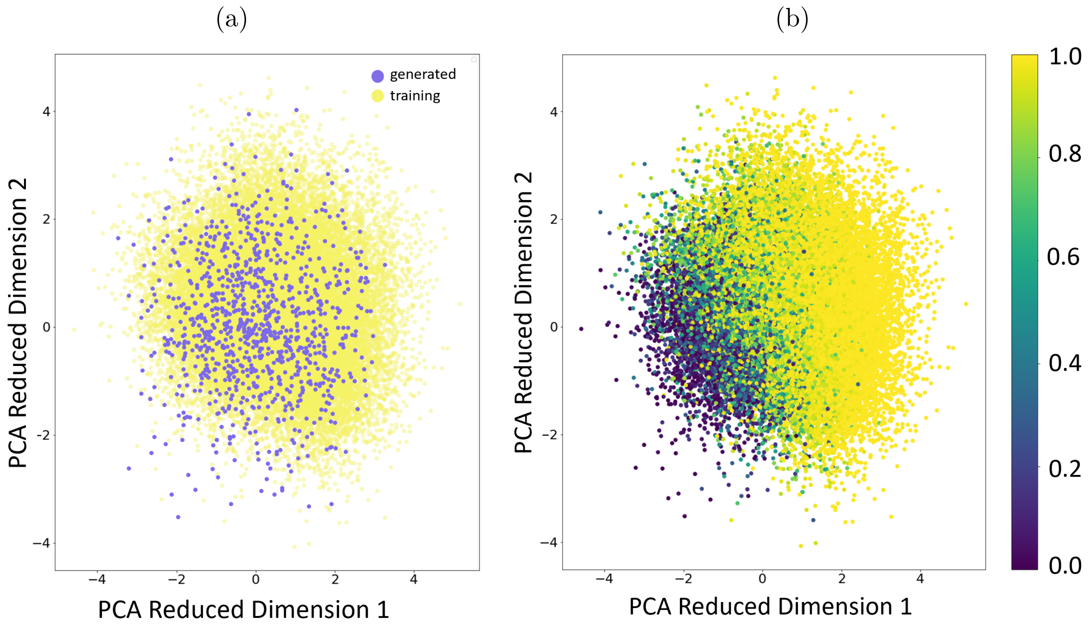


Figure S11: PCA of the latent space of the Cage-VAE, The PCA visualisation is coloured by a). Different data sources from generated and training b). Probability of shape-persistence mapped by the predictor. The colour bar shows the probability of the prediction on shape persistence where 0 and 1 are the lowest and highest probability of collapse, respectively. For clearer visualisation, only 30000 training molecules from the combined set of Original + Augmented dataset and all 1000 generated POCs were plotted.

## 5.4 Reconstruction

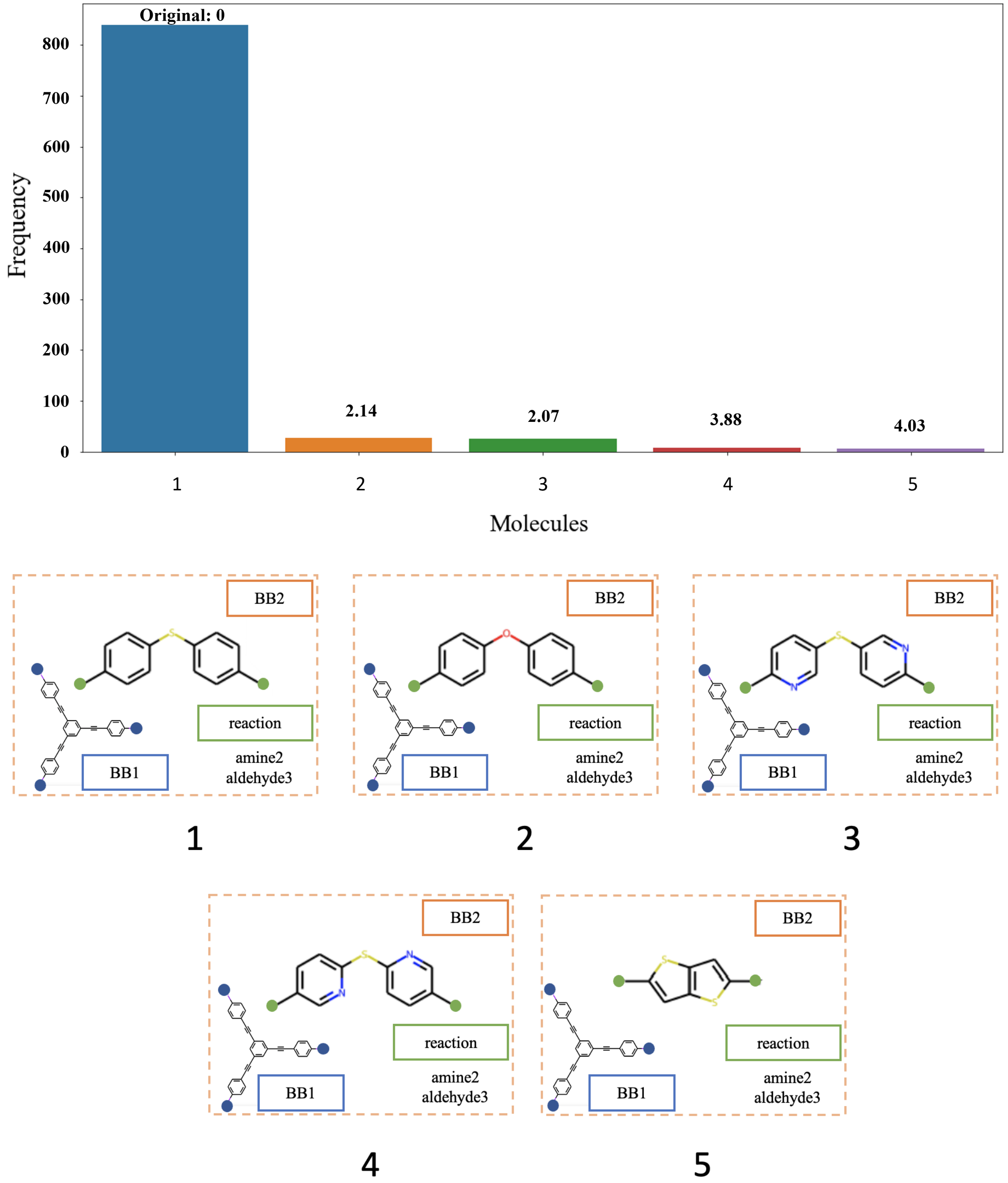


Figure S12: Histogram of 1000 reconstructions of a single POC in the latent space. The top five most frequent occurrences are displayed. The number above each bar indicates the distance from the point of the original input POC to the mean latent vectors of corresponding POCs in the latent space.

## 5.5 Interpolation

Linear interpolation (*lerp*) between latent vector $q_1$ and $q_2$ with interpolation factor $\mu$ can be described straightforwardly:

$$linear(q_1, q_2; \mu) = (1 - \mu)q_1 + \mu q_2 \tag{11}$$

Spherical linear interpolation (*slerp*) creates a circular arc with the claimed advantage of preventing the traversal of unlikely regions outside the learnt manifold.[S10] The formula was introduced as:[S11]

$$slerp(q_1, q_2; \mu) = \frac{sin(1 - \mu)\theta}{sin\theta} q_1 + \frac{sin\mu\theta}{sin\theta} q_2 \tag{12}$$

## 5.6 Filter

The filter is a flexible module that can be cascaded behind any generation method and uses a simple evaluation matrix to validate generated molecules with minimal computational cost. The filter has a layered structure that evaluates the quality of generated POCs in the order of validity, novelty, precursor validity and symmetry, as described in Section 5.1. Only the generated POC candidate that passes all layers of evaluations is considered valid output, otherwise, the generated candidate will be discarded and subsequently the generation method will be relaunched. The schematic diagram for the filter module is shown in Fig. S13.

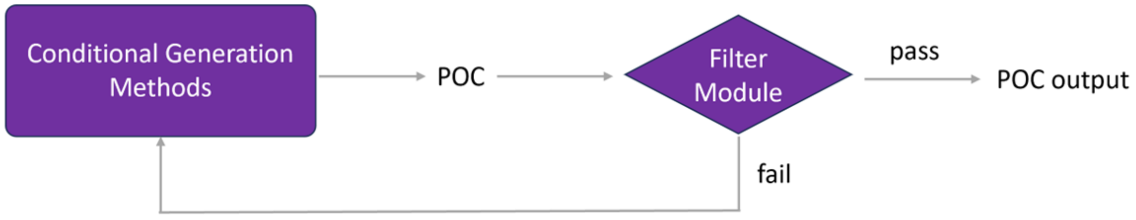


Figure S13: The schematic diagram of the filter module.

# References


(S1) Kingma, D. P.; Welling, M. Auto-Encoding Variational Bayes. *arXiv* **2013**, preprint, arXiv:1312.6114.

(S2) Goodfellow, I.; Bengio, Y.; Courville, A. *Deep Learning*; MIT press: Cambridge, 2016.

(S3) Fu, H.; Li, C.; Liu, X.; Gao, J.; Celikyilmaz, A.; Carin, L. Cyclical Annealing Schedule: A Simple Approach to Mitigating KL Vanishing. *arXiv* **2019**, preprint, arXiv:1903.10145.

(S4) Higgins, I.; Matthey, L.; Pal, A.; Burgess, C.; Glorot, X.; Botvinick, M.; Mohamed, S.; Lerchner, A. beta-VAE: Learning Basic Visual Concepts with a Constrained Variational Framework. International Conference on Learning Representations. 2017.

(S5) Burgess, C. P.; Higgins, I.; Pal, A.; Matthey, L.; Watters, N.; Desjardins, G.; Lerchner, A. Understanding Disentangling in $\beta$-VAE. *arXiv* **2018**, preprint, arXiv:1804.03599.

(S6) Gómez-Bombarelli, R.; Wei, J. N.; Duvenaud, D.; Hernández-Lobato, J. M.; Sánchez-Lengeling, B.; Sheberla, D.; Aguilera-Iparraguirre, J.; Hirzel, T. D.; Adams, R. P.; Aspuru-Guzik, A. Automatic Chemical Design Using a Data-Driven Continuous Representation of Molecules. *ACS Cent. Sci.* **2018**, *4*, 268–276.

(S7) Bowman, S. R.; Vilnis, L.; Vinyals, O.; Dai, A. M.; Jozefowicz, R.; Bengio, S. Generating Sentences from a Continuous Space. *arXiv* **2015**, preprint, arXiv:1511.06349.

(S8) Landrum, G. RDKit: Open-Source Cheminformatics Software. `http://www.rdkit.org`.

(S9) O'Boyle, N. M.; Banck, M.; James, C. A.; Morley, C.; Vandermeersch, T.; Hutchison, G. R. Open Babel: An open chemical toolbox. *Journal of Cheminformatics* **2011**, *3*, 1–14.

(S10) White, T. Sampling Generative Networks. *arXiv* **2016**, preprint, arXiv:1609.04468.

(S11) Shoemake, K. Animating Rotation with Quaternion Curves. Proceedings of the 12th Annual Conference on Computer Graphics and Interactive Techniques. New York, NY, USA, 1985; p 245–254.
